# CAV1-dependent mitochondrial transfer from hucMSCs reprograms epithelial lipid metabolism to relieve pulmonary fibrosis

**DOI:** 10.1186/s13287-026-05116-z

**Published:** 2026-06-19

**Authors:** Ye Shao, Jinjin Zhang, Hanchen Liu, Yujie Wang, Bo Liu, Xinglong Yuan, Mengqi Jiang, Changjun Lv, Songzi Zhang, Xiaodong Song, Hongbo Li

**Affiliations:** 1 Department of Respiratory and Critical Care Medicine, Shandong Medical and Pharmaceutical University Hospital, Shandong Key Lab of Complex Medical Intelligence and Aging, Shandong Medical and Pharmaceutical University, Yantai, 264003 China; 2 Department of Cellular and Genetic Medicine, Shandong Medical And Pharmaceutical University, No. 346, Guanghai Road, Yantai, 264003 Shandong China; 3 The Medical Basic Research Innovation Center of Airway Disease in North China, College of Basic Medical Sciences, Ministry of Education of China, Jilin University, Changchun, 130021 Jilin China; 4 Department of Cancer Center, the 970th Hospital of the People’s Liberation Army Joint Logistics Support Force, Yantai, 264000 Shandong China

**Keywords:** CAV1, hucMSCs, Mitochondrial transfer, Lipid metabolism, Pulmonary fibrosis

## Abstract

**Background:**

Idiopathic pulmonary fibrosis (IPF) is characterized by persistent epithelial injury accompanied by mitochondrial dysfunction. Although mesenchymal stem cells (MSCs) can restore epithelial function by donating mitochondria to damaged cells, the molecular mechanisms driving this process remain unclear. In this study, we demonstrate that caveolin-1 (CAV1) enhances mitochondrial transfer from human umbilical-cord-derived MSCs (hucMSCs) to injured epithelial cells.

**Methods:**

In vitro and in vivo bleomycin-induced models were used to evaluate mitochondrial transfer from hucMSCs to alveolar epithelial cells. Confocal microscopy and intravital lung imaging visualized mitochondrial transfer, while flow cytometry quantified transfer efficiency. Proteomic profiling, mitochondrial functional assays, and lipid analyses were conducted to explore CAV1-associated mechanisms and metabolic outcomes.

**Results:**

hucMSC treatment restored mitochondrial membrane potential, ATP production, and epithelial cell viability while reducing reactive oxygen species in injured MLE-12 cells. Proteomic analysis showed significant upregulation of CAV1 in hucMSCs cocultured with injured epithelial cells. In the same dataset, differentially expressed proteins were enriched in pathways related to cytoskeletal remodeling and vesicular transport, supporting a role for hucMSC membrane and trafficking dynamics in mitochondrial delivery. Functional validation confirmed that CAV1 overexpression markedly enhanced mitochondrial transfer and restored mitochondrial function, whereas CAV1 knockdown impaired both transfer efficiency and therapeutic outcomes. Mechanistically, transferred mitochondria promoted mitochondria–lipid droplet tethering, boosted fatty acid β-oxidation, and reduced lipid accumulation. CAV1-overexpressing hucMSCs alleviated alveolar epithelial injury and attenuated pulmonary fibrosis.

**Conclusions:**

Our findings identify CAV1 as a crucial mediator of hucMSC-mediated mitochondrial transfer, which enhances epithelial repair through mitochondrial donation and metabolic reprogramming. These insights provide a mechanistic foundation for optimizing stem cell-based therapies in pulmonary fibrosis.

**Graphical Abstract:**

**Figure:**
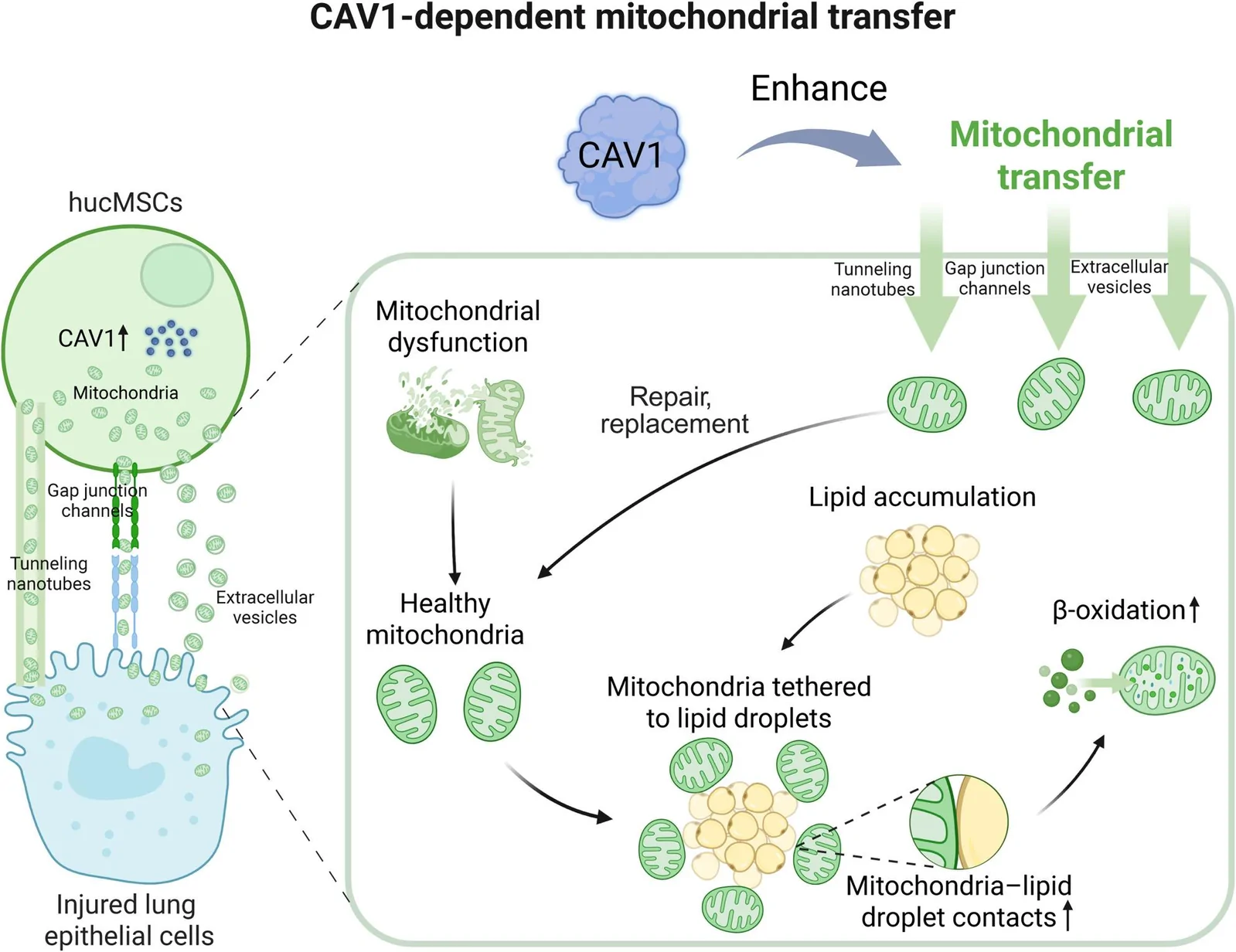

**Supplementary Information:**

The online version contains supplementary material available at https://doi.org/10.1186/s13287-026-05116-z.

## Background

Idiopathic pulmonary fibrosis (IPF) is a chronic and progressive lung disease that leads to high mortality, with a median survival of just three years after diagnosis [1, 2]. Recent research suggests that IPF’s pathogenesis is closely associated with abnormal regeneration of alveolar epithelial cells (AECs) [3, 4]. Repeated injury to these cells disrupts the repair process, which in turn activates fibroblasts [5, 6]. These cells migrate and proliferate, resulting in excessive extracellular matrix (ECM) deposition and a remodeling of lung tissue [7]. Currently, the only FDA-approved treatments for IPF are pirfenidone and nintedanib. Although these drugs target fibroblast activity, their efficacy is limited due to poor drug delivery to the lungs and suboptimal promotion of repair following epithelial injury [8, 9]. Therefore, developing new therapeutic strategies that address the early stages of IPF pathology is crucial for improving clinical outcomes.

Mesenchymal stem cell (MSC)-based therapies have emerged as a promising treatment approach for pulmonary diseases. Among the various sources of MSCs, human umbilical cord-derived MSCs (hucMSCs) have gained significant attention due to their low immunogenicity, strong immunomodulatory and anti-inflammatory properties, and enhanced regenerative potential. Compared with MSCs from other tissue sources, hucMSCs display superior proliferative and differentiation capabilities, leading to improved therapeutic efficacy in pulmonary fibrosis [10]. Preclinical studies have shown that hucMSCs can attenuate pulmonary fibrosis by modulating inflammation and promoting tissue repair [11]. Moreover, hucMSC-derived extracellular vesicles have also demonstrated therapeutic potential in pulmonary fibrosis in both experimental models and clinical studies [12]. Despite these advantages, the exact mechanisms underlying their therapeutic effects remain unclear. Additionally, exposure to toxic and oxidative environments significantly reduces the survival of transplanted hucMSCs, which typically decline in viability within 24 h after infusion [13]. Their limited self-renewal capacity and short lifespan further restrict their therapeutic potential. Therefore, understanding how hucMSCs work and developing strategies to enhance their efficacy is essential.

Recent evidence highlights that mitochondrial dysfunction in AECs, characterized by a reduction in functional mitochondria and impaired clearance of damaged ones, plays a pivotal role in the development of IPF [14, 15]. Disruptions to mitochondrial homeostasis not only mark the progression of the disease but also act as a driving force behind fibrosis. The imbalance between functional and dysfunctional mitochondria leads to insufficient ATP production and excessive reactive oxygen species (ROS), impairing epithelial cell function and accelerating the development of IPF [16]. Restoring mitochondrial function in these cells is therefore critical for effective lung repair. Recently, mitochondrial transfer has emerged as a promising therapeutic approach. MSCs can transfer mitochondria to recipient cells through tunneling nanotubes (TNTs), cell fusion, or extracellular vesicles, helping to restore cellular function [17]. However, challenges remain, including limited mitochondrial biogenesis, poor transfer efficiency, and rapid depletion of transferrable mitochondria, all of which hinder the effectiveness of mitochondrial supplementation [18]. Emerging evidence suggests that these limitations are closely associated with the structural and functional integrity of donor cell membranes and their capacity for cytoskeletal remodeling and vesicular trafficking, which are critical for successful intercellular mitochondrial exchange [19, 20]. Caveolin-1 (CAV1), a membrane-associated scaffolding protein that regulates caveolae formation and intercellular communication, has been implicated in both mitochondrial function and vesicular transport [21, 22]. However, it is not yet clear whether CAV1 plays a role in facilitating mitochondrial transfer from hucMSCs to injured epithelial cells. This study aims to determine whether CAV1 regulates the ability of hucMSCs to transfer mitochondria and to explore the potential role of this process in restoring metabolic homeostasis in epithelial cells during pulmonary fibrosis.

## Materials and methods

### Isolation and culture of hucMSCs

Human umbilical cord–derived mesenchymal stem cells (hucMSCs) were isolated from the Wharton’s jelly of an umbilical cord obtained from a healthy full-term pregnancy delivered naturally. Written informed consent was obtained from the donor. The study protocol was approved by the Medical Ethics Committee of Binzhou Medical University (Approval No. 2024-L086; approval date: March 11, 2024). Cells used in this study were derived from a single donor. Fresh human umbilical cords were collected and thoroughly rinsed with phosphate-buffered saline (PBS) to remove surface contaminants, followed by disinfection in 75% ethanol. The remaining blood vessels and connective tissue were carefully removed, and the umbilical cord was cut into small fragments of approximately 1 mm³. These fragments were evenly placed in 10 cm culture dishes. The explants were cultured in Dulbecco’s modified Eagle medium: Nutrient Mixture F-12 (DMEM/F12) supplemented with 10% fetal bovine serum (FBS) and 100 U/ml penicillin-streptomycin, and incubated at 37 °C in a humidified atmosphere of 5% CO_2_ and 95% air. After 7 days, cells were observed migrating out from the tissue fragments. The tissue fragments were removed around day 14. When the adherent cells reached approximately 90% confluence (passage 0, P0), they were digested using 0.25% trypsin-EDTA and seeded at a density of 5 × 10^5^ cells per dish for subculture.

### Characterization of hucMSCs surface markers

Passages 3 to 5 of hucMSCs, which were approximately 90% confluent, were harvested, and 1 × 10⁶ cells were added to each tube. After centrifugation, the supernatant was discarded and the cell pellets were resuspended in 1× PBS. The following antibodies (all from BioLegend) were added: CD34, CD45, HLA-DR, CD90, CD105, CD44, and CD73. The samples were incubated in the dark at 4 °C for 30 min. After incubation, 1 mL of PBS was added to each tube, and antibody expression was analyzed using flow cytometry (Becton, Dickinson and Company).

### Adipogenic, osteogenic and chondrogenic differentiation

The trilineage differentiation potential of hucMSCs was assessed through Oil Red O staining for lipid droplet (LD) formation (adipogenesis), Alizarin Red S staining for mineralized matrix deposition (osteogenesis), and Alcian Blue staining for proteoglycan-rich extracellular matrix (chondrogenesis). Adipogenic, osteogenic, and chondrogenic differentiation were performed using the HyCyte Human Umbilical Cord Marrow Mesenchymal Stem Cells Adipogenic Differentiation Kit, Osteogenic Differentiation Kit, and Chondrogenic Differentiation Kit (Cas9X), respectively. Briefly, 4 × 10⁴/mL hucMSCs were seeded on 24-well plates. When the cell density reached approximately 95%, the supernatant was discarded and replaced with the respective differentiation media. The culture medium was exchanged every 3 days. After 20 days, Oil Red O, Alizarin Red, and Alcian Blue staining were performed, and the results were observed under a microscope.

### Conditioned medium collection of hucMSCs

hucMSCs were seeded at 1 × 10^5^ cells/dish and cultured in DMEM/F12 complete medium. Once the cells attached, they were washed three times with PBS and incubated in serum-free DMEM to stimulate the release of secreted mitochondria. After 24 h of incubation, the culture medium was filtered through a 1.2-µm syringe filter to remove cellular debris, resulting in the hucMSC-conditioned medium (Mt-HCM) containing the secreted mitochondria. To obtain mitochondria-depleted medium (Md-HCM), Mt-HCM was further filtered through a 0.22-µm syringe filter to effectively remove the mitochondria. The mitochondrial activity markers in both Mt-HCM and Md-HCM were analyzed to confirm that Mt-HCM contained biologically active mitochondria, whereas Md-HCM served as a negative control.

### Establishment of epithelial cell injury model

The SV40T-immortalized MLE-12 cell line, which retains stable alveolar epithelial type II (AEC-II) characteristics, was purchased from Cellverse Co., Ltd. (Shanghai, China). These cells were maintained in DMEM/F12 (SparkJade, Shandong, China), a 1:1 mixture of DMEM and Ham’s F-12, combined with high glucose, trace elements, and hormones, making it suitable for culturing epithelial cells and MSCs. The medium was supplemented with 10% FBS (SparkJade) and 100 U/ml penicillin-streptomycin (SparkJade), and the cells were incubated at 37 °C in a humidified incubator with 5% CO_2_ and 95% air. To establish the epithelial injury model, MLE-12 cells were assigned to the following experimental groups: Normal, BLM, and treatment groups involving coculture with hucMSCs. In the BLM group, MLE-12 cells were exposed to bleomycin (BLM; 10 µg/mL) for 24 h to induce epithelial injury. For all treatment groups, MLE-12 cell samples used for downstream analyses were obtained after 24 h of BLM treatment, 24 h of coculture with hucMSCs, and subsequent flow cytometric sorting. The detailed coculture and sorting strategies are described below.

### Lentiviral vector construction and transduction

To label mitochondria, a lentiviral vector encoding mitochondria-targeted DsRed (pHBLV-CMV-Mito-DsRed-3×FLAG-PGK-Puro-WPRE3; Hanbio Co., Ltd., Shanghai, China) was used. This construct contains a mitochondrial targeting sequence (MTS) derived from cytochrome c oxidase subunit VIII (COX8), ensuring specific localization of DsRed to the mitochondrial matrix. Cells were transduced at a multiplicity of infection (MOI) of 15. All other lentiviral constructs were obtained from Keyybio Co., Ltd. (Shandong, China). For cytoplasmic fluorescent labeling, MLE-12 cells and hucMSCs were transduced with a ZsGreen-expressing lentiviral vector (VP157-CMV-MCS-EF1-ZsGreen-T2A-Puro). The MOI was set at 5 for MLE-12 cells and 15 for hucMSCs. For CAV1 overexpression, the coding sequence of CAV1 was cloned into the multiple cloning site (MCS) of the VP157-CMV-MCS-EF1-Puro vector under the control of the CMV promoter. A fluorescent version (VP157-CMV-MCS-EF1-ZsGreen-T2A-Puro) was used when required. For gene silencing, CAV1-targeting shRNA was cloned into the pLenti-U6-shRNA-CMV-Puro vector, in which shRNA expression was driven by the U6 promoter and puromycin resistance was controlled by the CMV promoter. A fluorescent version (pLenti-U6-shRNA-EF1-ZsGreen-CMV-Puro) was used when required. The target sequences for CAV1 knockdown were as follows: sense, GCGAGAAGCAAGTGTACGATT; antisense, TCGTACACTTGCTTCTCGCTT. For lentiviral transduction, cells were incubated with viral particles in complete medium supplemented with polybrene (5 µg/mL) to enhance transduction efficiency. After 24 h, the medium was replaced with fresh complete medium. At 48 h post-transduction, cells were subjected to puromycin selection (2 µg/mL) to establish stable transduced cell lines. Selection was continued until all untransduced cells were eliminated, yielding a nearly homogeneous transduced population.

### Fluorescent labeling and subsequent coculture of hucMSCs and MLE-12 cells

To distinguish donor and recipient cells and to evaluate mitochondrial transfer, hucMSCs and MLE-12 cells were fluorescently labeled using lentiviral vectors carrying reporter genes targeted either to the mitochondria or the cytoplasm, thereby avoiding the non-specific intercellular transfer that may occur with exogenous dyes. For mitochondrial transfer experiments, hucMSCs were transduced with a mitochondria-targeted DsRed reporter (mtDsRed; HanBio, China), whereas MLE-12 cells were transduced with a cytoplasmic ZsGreen reporter (Keyybio, China). After transduction, both cell types were maintained under standard culture conditions and used for subsequent coculture experiments. In this setting, ZsGreen-expressing MLE-12 cells served as recipient cells, whereas mtDsRed-labeled hucMSCs served as donor cells.

Unless otherwise specified, coculture-based experiments were performed using an MLE-12: hucMSC ratio of 3:1. The total seeding density was maintained at 1.5 × 10^5^ cells/cm², and the absolute cell number was adjusted according to the surface area of the culture vessel to ensure a confluence of approximately 80%–90% under coculture conditions. These coculture conditions were applied consistently across coculture-based assays, including mitochondrial transfer analysis and cell-sorting experiments.

Depending on the downstream application, cocultures were established in 10-cm culture dishes for cell sorting or in glass-bottom confocal dishes for live-cell imaging.

### Observations of mitochondrial transfer from hucMSCs to MLE-12 cells

Mitochondrial transfer from hucMSCs to MLE-12 cells was observed using confocal laser scanning microscopy. MLE-12 cells expressing cytoplasmic ZsGreen and hucMSCs labeled with mitochondrial DsRed were co-seeded onto glass-bottom confocal dishes for imaging at an MLE-12:hucMSC ratio of 3:1, with a total seeding density of 1.5 × 10^5^ cells/cm². After 24 h of coculture, the cells were then gently washed three times with PBS and immediately imaged using a Zeiss LSM880 confocal microscope. Fluorescence images were captured using appropriate excitation and emission channels for ZsGreen and DsRed.

### Quantitative analysis of mitochondrial transfer rate

After 24 h of coculture, cells were harvested by trypsin-EDTA digestion to quantify the mitochondrial transfer from hucMSCs to MLE-12 cells. The cell suspension was gently washed twice with PBS to remove residual enzymes and debris, then resuspended in PBS for flow cytometric analysis using the LSRFortessa™ system (BD Biosciences, USA). During the analysis, ZsGreen-positive MLE-12 cells were gated based on their green fluorescence to accurately identify the recipient cell population. Within this gate, cells also positive for red fluorescence (mtDsRed) were considered to have received mitochondria from hucMSCs. The mitochondrial transfer rate was calculated as the percentage of ZsGreen⁺/mtDsRed⁺ double-positive cells among total ZsGreen⁺ cells. Proper single-color controls (ZsGreen-MLE-12 and mtDsRed-hucMSCs alone) were included to account for spectral overlap and autofluorescence. Flow cytometry data were analyzed using FlowJo software, and the mitochondrial transfer efficiency was represented as the proportion of double-positive cells in bar graphs.

### Isolation of ZsGreen-MLE-12 and mtDsRed-hucMSCs from the coculture system

To distinguish donor and recipient cell populations in the coculture system, fluorescence-activated cell sorting (FACS) was performed based on cytoplasmic ZsGreen expression in MLE-12 cells and mitochondrial DsRed expression in hucMSCs. Unless otherwise specified, coculture-based experiments were performed using an MLE-12:hucMSC ratio of 3:1. The total seeding density was maintained at 1.5 × 10^5^ cells/cm², and the absolute cell number was adjusted according to the surface area of the culture vessel. After 24 h of coculture, cells were harvested by trypsin-EDTA digestion, washed twice with PBS to remove residual enzymes and debris, and resuspended for sorting using a CytoFLEX SRT cell sorter (Beckman Coulter, USA). ZsGreen fluorescence was excited with a 488-nm blue laser and detected in the FL1 channel, whereas DsRed fluorescence was excited with a 561-nm yellow-green laser and detected in the FL3 channel. Single-color controls (ZsGreen-MLE-12 and mtDsRed-hucMSCs cultured separately) were used to define fluorescence boundaries and minimize spectral overlap during gating. For most downstream analyses, including mitochondrial transfer quantification, proteomic profiling, and functional assays, all ZsGreen-positive cells were defined as the MLE-12 population, irrespective of their DsRed signal. In contrast, ZsGreen-negative cells were considered the hucMSC population and were collected separately for proteomic analyses. For fluorescence-based downstream experiments, such as immunofluorescence imaging and flow-cytometric functional assays, an alternative sorting strategy was employed to avoid potential interference from fluorescent reporters. In these experiments, unlabeled MLE-12 cells were cocultured with ZsGreen-labeled hucMSCs, and ZsGreen-negative cells were collected as the MLE-12 population for subsequent analyses. The detailed gating strategy used for cell identification and sorting is illustrated in Supplementary Fig. 3, where the sequential gating steps and the definition of sorted populations are presented.

### Animal model

All animal experiments were conducted and reported in accordance with the ARRIVE 2.0 guidelines and were approved by the Animal Ethics Committee of Binzhou Medical University (Approval No. 2024-L087). Eight-week-old male C57BL/6 mice (20 ± 5 g) were purchased from Jinan Pengyue Experimental Animal Breeding Co., Ltd. (China) and housed under specific pathogen-free (SPF) conditions (22 ± 2 °C, 12-h light/dark cycle) with free access to food and water. Mice were randomly assigned to the following experimental groups (*n* = 10 per group): Sham, BLM, BLM + hucMSCs, BLM + hucMSCs (Mock), BLM + hucMSCs (Over), and BLM + hucMSCs (shRNA). All investigators responsible for data collection and analysis were blinded to group allocation throughout the study. Mice were anesthetized by intraperitoneal injection of 2.5% Avertin (tribromoethanol; 250 mg/kg) and administered BLM (5 mg/kg) via intratracheal spraying using a Penn-Century MicroSprayer (Penn-Century Inc.) to induce pulmonary fibrosis. Sham mice received an equal volume of sterile saline using the same procedure. Twenty-four hours later, mice were treated via tail vein injection with 1 × 10⁶ hucMSCs in 100 µL PBS, including untreated hucMSCs, hucMSCs carrying an empty vector (Mock), a CAV1-overexpression construct (Over), or a CAV1-knockdown construct (shRNA). Twenty-eight days after BLM administration, mice were deeply anesthetized with 2.5% Avertin (tribromoethanol; 250 mg/kg, intraperitoneally) and subsequently euthanized by cervical dislocation. Death was verified by the absence of cardiac activity, reflex responses, and spontaneous breathing. Humane endpoints were strictly applied to minimize animal suffering. Mice exhibiting > 20% body-weight loss or signs of severe respiratory distress were euthanized immediately under deep anesthesia.

### Intravital pulmonary imaging

Intravital lung imaging was supported by IVIM Technology. To label alveolar epithelial cells, an adeno-associated viral vector (pCAAV-SP-C-ZsGreen1-WPRE) driven by the SP-C promoter was intratracheally administered at ~ 1 × 10¹¹ Vg per mouse. After 3–4 weeks, pulmonary fibrosis was induced by bleomycin (BLM). Twenty-four hours later, DsRed-labeled hucMSCs (1 × 10⁶ cells in 0.1 mL PBS) were injected via the tail vein. Intravital imaging was conducted 24 h after cell infusion to visualize mitochondrial transfer. Mice were anesthetized with 1% pentobarbital sodium (10 mL/kg, i.p.) and mechanically ventilated (RV-01, Kent Scientific) with 24–30 mmHg inspiratory pressure, 120–130 breaths/min rate, and 2 cm H₂O PEEP. Body temperature was maintained at 37 °C using the integrated temperature control module (IVM Temp Module, IVIM Technology). For imaging, mice were placed in the right-lateral position and the left thorax was opened between the 3rd and 4th ribs. A lung imaging window (IVIM Lung Imaging Window Set) was applied to the lung surface and stabilized using 20–30 mmHg negative pressure (NVC 2300a, EYELA). Images were acquired under identical settings and reconstructed with IVM Studio software.

### hucMSC-targeted lung experiment

To assess the in vivo pulmonary localization of transplanted hucMSCs, cells were labeled using a DiI Cell Plasma Membrane Staining Kit (Beyotime, China) according to the manufacturer’s instructions. Briefly, hucMSCs were incubated with DiI working solution at 37 °C for 20 min, followed by washing three times with PBS to remove unbound dye. The cells were then collected by centrifugation at 161 × g for 5 min, resuspended in serum-free medium at a final concentration of 1.0 × 10^6^ cells/mL, and administered to mice via tail vein injection. Lung tissues were collected at 1, 3, 7, and 14 days after transplantation. Frozen lung sections were prepared using a cryostat microtome (Leica CM1950, Germany) and imaged under a fluorescence microscope to evaluate the distribution of transplanted hucMSCs.

### Micro-CT measurement

After anesthetizing the mice with 2.5% Avertin (0.02 mL/g body weight, intraperitoneally), they were placed on a scanner bed for in vivo Micro-CT imaging (PerkinElmer). The X-ray settings were 90 kV and 88 µA. The CT images had a field of view of 36 mm and an exposure duration of 4 min. Two-dimensional tomographic images were reconstructed using SimpleViewer imaging software (SimpleViewer), and three-dimensional reconstructions were performed with Analyze 11.0 (AnalyzeDirect).

### Pulmonary function analysis

Mice were anesthetized by intraperitoneal injection of 0.02 mL/g of 2.5% Avertin. A small incision was made in the trachea, and endotracheal intubation was performed using a plastic intravenous catheter connected to a metal cannula inserted into a small hole in the mouse trachea. The catheter was connected to a pulmonary ventilator (DSI Buxco, USA). The mechanical ventilation settings included a respiratory rate of 150 breaths/min, a tidal volume of 10 mL/kg, and PEEP set at 3 cm H_2_O. A negative pressure-driven forced expiratory (NPFE) maneuver was applied, inflating the lungs to a pressure of 30 cm H_2_O for 2 s, followed by connection to a negative pressure reservoir (-50 cm H_2_O) for 2 s. Forced vital capacity (FVC) was calculated from the flow-volume loop generated during pulmonary deflation.

### H&E and Masson’s trichrome staining

Lung tissues were fixed in 4% paraformaldehyde for 24 h, then dehydrated and infiltrated with paraffin overnight. The tissues were embedded in paraffin and sectioned into 4 μm slices using a Leica microtome (Leica, Germany). The paraffin sections were dewaxed and stained with either hematoxylin and eosin (H&E) or Masson’s trichrome staining kits (Solarbio, China). Histological evaluation of the lung sections from each group was performed under a light microscope (Olympus, Japan).

### Cell viability assay

Cell viability was assessed using a Cell Counting Kit-8 (CCK-8; Meilun Biotechnology, Dalian, China) according to the manufacturer’s instructions. MLE-12 cells were seeded into 96-well plates at a density of 6 × 10^3^ cells per well. For experiments involving co-culture, MLE-12 cells were first co-cultured with hucMSCs as described above and subsequently isolated by fluorescence-activated cell sorting (FACS) based on ZsGreen expression. The sorted MLE-12 population was then seeded into 96-well plates at 6 × 10^3^ cells per well. After treatment, the culture medium was replaced with fresh medium containing 10% CCK-8 reagent and incubated for 2 h at 37 °C. Absorbance was measured at 450 nm using a microplate reader, and cell viability was calculated according to the manufacturer’s instructions.

### Immunofluorescence analysis of surfactant protein C (SPC) expression

MLE-12 cell samples from the indicated groups were seeded at a density of 1 × 10^5^ cells/mL on sterilized glass coverslips in 24-well plates; for treatment groups, the MLE-12 population was obtained according to the coculture and sorting procedures described above. After attachment, cells were washed three times with 1× PBS, fixed with 4% paraformaldehyde for 30 min at room temperature, and permeabilized with 0.3% Triton X-100 for 15 min. Non-specific binding was blocked by incubating the cells with goat serum for 30 min at room temperature. Cells were then incubated overnight at 4 °C with a primary antibody against SPC. The following day, cells were rewarmed at 37 °C for 30 min, then incubated with a fluorescently labeled secondary antibody for 50 min at room temperature. After washing with PBS, nuclei were counterstained with 0.25% DAPI solution (200 µL per well) for 5 minutes, followed by three washes with PBS. Finally, an antifade mounting medium was applied to the coverslips, and fluorescence images were captured using a Zeiss LSM880 confocal laser scanning microscope.

### Measurement of mitochondrial membrane potential (MMP)

MMP in MLE-12 cells was assessed using a JC-1 assay kit (Beyotime, China). MLE-12 cell samples from the indicated groups were collected for analysis, with 3 × 10⁶ cells per group. For the treatment group, the MLE-12 population was obtained according to the coculture and sorting procedures described above. After washing twice with pre-chilled PBS, the cells were resuspended in JC-1 working solution and incubated at 37 °C for 20 min in the dark. After incubation, the cells were washed twice with JC-1 staining buffer and analyzed immediately by flow cytometry (BD Canto II, USA). The monomeric form of JC-1 was detected in the FL1 channel, and the aggregated form of JC-1 was detected in the FL2 channel. To assess MMP in Mt-HCM and Md-HCM, the conditioned media were mixed with 5× diluted JC-1 working solution at a 1:1 ratio and incubated at 37 °C in the dark for 30 min. After incubation, fluorescence intensity was measured using a multimode microplate reader. The ratio of red fluorescence (J-aggregates, 590 nm) to green fluorescence (JC-1 monomers, 530 nm) was calculated to evaluate changes in MMP.

### Determination of ATP content

Approximately 1 × 10⁶ MLE-12 cell samples from the indicated groups were harvested and washed twice with pre-chilled PBS (4 °C). For the treatment group, the MLE-12 population was obtained according to the coculture and sorting procedures described above. Cells were then lysed in 200 µL of ice-cold ATP lysis buffer and incubated on ice for 15 min. The lysates were centrifuged at 12,000 × g for 10 min at 4 °C, and the supernatants were collected. For extracellular samples, the culture medium was collected, centrifuged at 2000 × g for 10 min, and the supernatant was further centrifuged at 20,000 × g for 20 min at 4 °C. After sample collection, ATP levels were measured using an ATP assay kit (Beyotime, China). The prepared sample solutions were mixed with an equal volume of ATP detection working solution and incubated for 30 min at room temperature in opaque-walled 96-well plates. Chemiluminescence intensity was measured using a multifunctional microplate reader. ATP concentrations were calculated based on a standard curve and normalized to total protein content.

### Intracellular ROS determination

Intracellular ROS levels in MLE-12 cells were measured using a Reactive Oxygen Species Assay Kit (Beyotime, China). MLE-12 cell samples from the indicated groups were collected and incubated with a 50 µM working solution of DCFH-DA. For the treatment group, the MLE-12 population was obtained according to the coculture and sorting procedures described above. The cells were incubated at room temperature for 30 min, then washed three times with PBS and harvested for quantitative analysis by flow cytometry.

### Western blot

Cells or lung tissues were harvested and lysed in RIPA buffer supplemented with protease and phosphatase inhibitors. The samples were then centrifuged at 12,000 rpm at 4 °C for 5 min to collect the supernatant. Protein concentrations were measured using a bicinchoninic acid protein assay kit (Coolaber, China). Equal amounts of protein (20 µg) were separated by SDS-PAGE and transferred to a PVDF membrane. After blocking the PVDF membrane in 5% skim milk for 2 h at room temperature, it was incubated with primary antibodies overnight at 4 °C with shaking, followed by incubation with secondary antibodies at room temperature for 1 h. Western blot signals were detected and scanned using the Tanon 5200 Multi (ThermoFisher Scientific, USA). The primary antibodies used were as follows: anti-collagen I (1:1000, Affinity, China), anti-*α-SMA* (1:1000, Bioswamp, China), anti-Vimentin (1:1000, Bioswamp, China), anti-GAPDH (1:10,000, Bioswamp, China), anti-SPC (1:1000, Bioswamp, China), anti-HSL (1:1000, Bioswamp, China), anti-ATGL (1:1000, Bioswamp, China), and anti-ACOT1 (1:1000, Bioswamp, China). The secondary antibodies used were goat anti-mouse HRP-conjugated IgG (1:7000, Bioswamp, China) and goat anti-rabbit HRP-conjugated IgG (1:7000, Bioswamp, China).

### Proteomic analyses of hucMSCs and MLE-12 cell samples

For the proteomic analysis of recipient epithelial cells shown in Fig. 3, the experimental groups were defined as Normal, BLM, and BLM + hucMSCs. The actual samples subjected to proteomic analysis were the corresponding MLE-12 cell populations from each group. In the coculture condition, ZsGreen-positive MLE-12 cells were sorted from the coculture system and used for protein extraction. For the proteomic analysis of donor hucMSCs (Fig. 5), the corresponding hucMSC population was sorted separately according to the gating strategy described above. Mixed coculture lysates were not used for proteomic analysis. After extracting and purifying the cellular proteins, the samples were quantified with a bicinchoninic acid (BCA) assay kit. The proteins were then reduced with 100 mM dithiothreitol at 56 °C for 1 h and alkylated with 500 mM iodoacetamide at room temperature for 1 h, in the dark. The protein samples were digested overnight at 37 °C with proteomics-grade trypsin. The resulting peptides were separated using a NanoElute high-performance liquid chromatography (HPLC) system, ionized, and analyzed by tandem mass spectrometry (MS/MS) on a Q Exactive Plus instrument (Thermo Fisher Scientific). The MS data were processed using Proteome Discoverer (version 2.4.0.305). Protein annotation and classification were performed through Gene Ontology (GO) analysis using the UniProt-GOA database (https://www.ebi.ac.uk/GOA/). Pathway analysis and molecular interaction networks were constructed based on the Kyoto Encyclopedia of Genes and Genomes (KEGG) database.

### Statistical analysis

Data were analyzed using GraphPad Prism 9.0 statistical software and are expressed as mean ± SD. Comparisons between two groups were performed using t-tests, whereas comparisons among multiple groups were made using one-way analysis of variance (one-way ANOVA) followed by Newman-Keuls post-hoc tests. Statistical significance was set at *P* < 0.05.

## Results

### Identification of hucMSCs

**Fig. 1:**
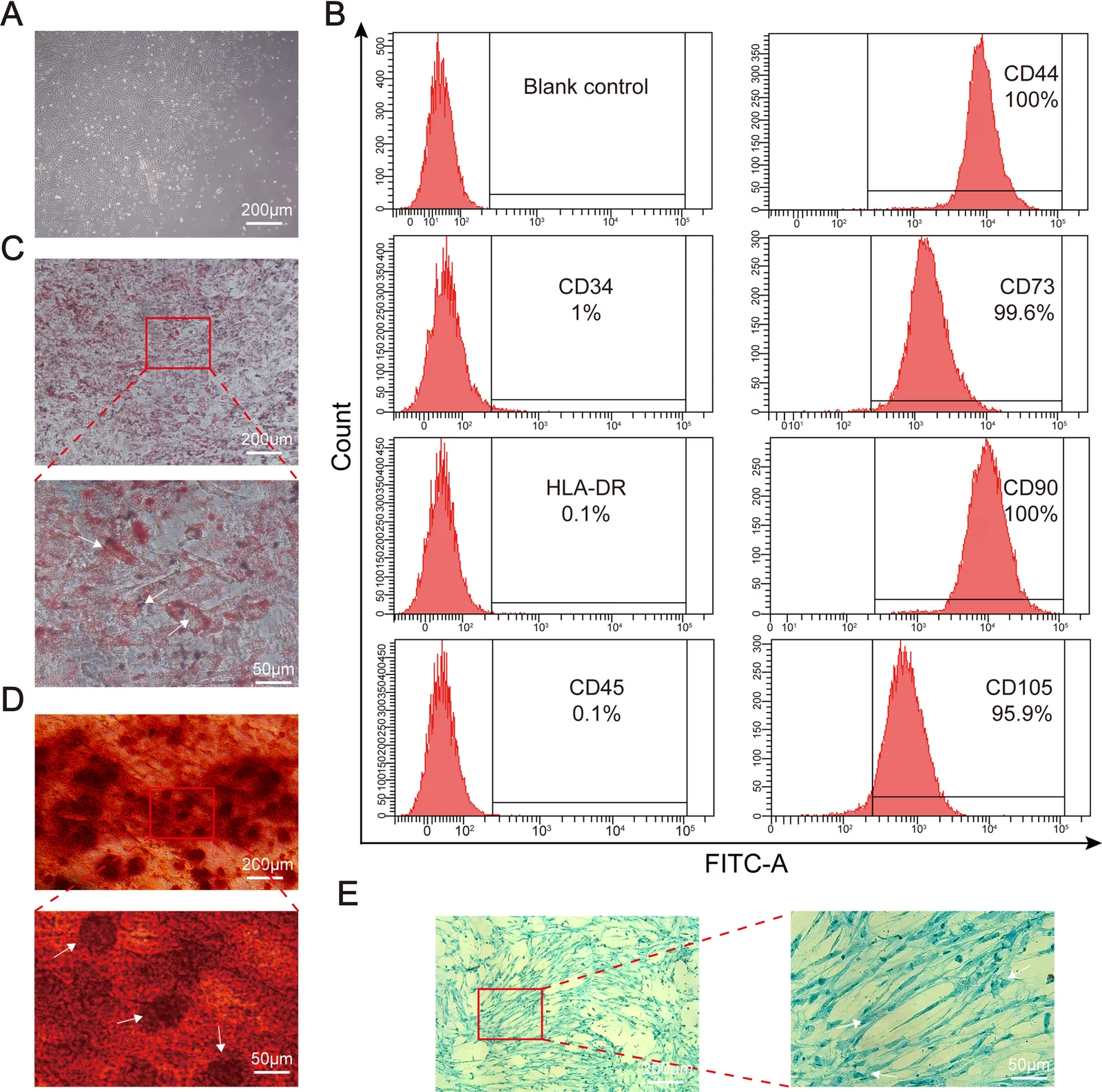
Identification of hucMSCs. **A** hucMSCs exhibited a long, spindle-shaped morphology under an inverted microscope, arranged either in parallel or in a whirlpool pattern. **B** Flow cytometry analysis of cell surface markers showed that hucMSCs highly express CD73, CD90, CD44, and CD105, whereas lacking expression of HLA-DR, CD34, and CD45. **C** Oil Red O staining demonstrated adipogenic differentiation by showing LD accumulation in hucMSCs. **D** Alizarin Red staining confirmed osteogenic differentiation, revealing bone matrix formation. **E** Alcian Blue staining identified chondrogenic differentiation by the presence of proteoglycan-rich extracellular matrix

After isolating cells from human umbilical cords, we identified them as hucMSCs based on their morphology, ability to differentiate into osteogenic, adipogenic, and chondrogenic lineages, and expression of specific surface markers. The cells displayed a long, spindle-shaped morphology, often arranged in parallel or in a whirlpool-like pattern (Fig. 1A). Flow cytometry analysis confirmed the positive expression of CD105, CD90, CD73, and CD44, and the negative expression of HLA-DR, CD34, and CD45 (Fig. 1B). Adipogenic differentiation was confirmed by Oil Red O staining, which revealed the accumulation of red lipid droplets (LDs) (Fig. 1C). Alizarin Red staining indicated successful osteogenic induction and mineralization (Fig. 1D), while Alcian Blue staining confirmed chondrogenic differentiation (Fig. 1E). These results collectively validate the successful isolation and characterization of hucMSCs.

### hucMSCs treatment improves injured AECs and attenuates pulmonary fibrosis

**Fig. 2:**
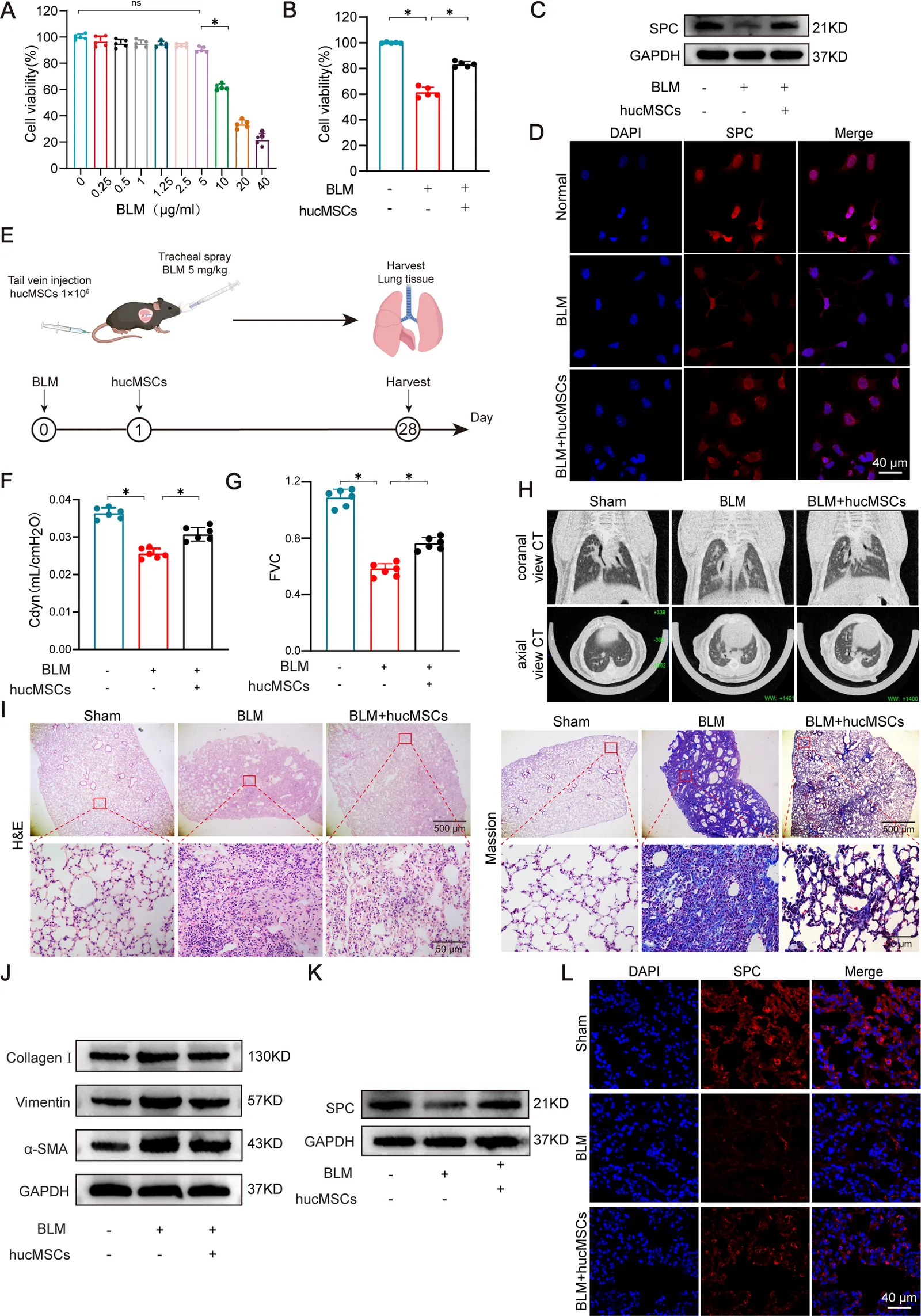
hucMSCs alleviated BLM-induced epithelial cells injury in vitro and in vivo. **A** MLE-12 cells were exposed to various concentrations of BLM for 24 h. CCK-8 assays showed that 10 µg/mL BLM significantly reduced cell viability. **B** hucMSC treatment restored cell viability in BLM-injured MLE-12 cells. **C** Western blot analysis showed that SPC expression was upregulated in the hucMSC-treated group compared to the BLM group. **D** Immunofluorescence staining revealed reduced SPC fluorescence intensity in the BLM group, which was restored after hucMSC treatment. **E** Mice were treated with BLM via intratracheal instillation to induce pulmonary fibrosis, followed by intravenous injection of hucMSCs through the tail vein for therapeutic intervention. **F** Cdyn was significantly decreased in BLM-treated mice and restored after hucMSC treatment. **G** FVC was reduced in the BLM group but improved following hucMSC administration. **H** Micro-CT images showed reduced fibrotic lesions and improved lung architecture after hucMSC treatment. **I** H&E staining demonstrated thickened alveolar walls and inflammatory infiltration in the BLM group, which were alleviated by hucMSC treatment. Masson’s trichrome staining revealed reduced collagen deposition in the lungs of hucMSC-treated mice. **J** Western blot analysis showed reduced expression of fibrosis-related proteins, such as collagen I, vimentin, and α-SMA, in the hucMSC-treated group compared to the BLM group. **K** SPC levels were reduced in BLM-treated mice and restored after hucMSC treatment. **L** Immunofluorescence staining showed a reduction in SPC expression in the BLM group, which was restored following hucMSC treatment. Data are presented as mean ± SD, *n* = 6, *P < 0.05

To assess the therapeutic effects of hucMSCs on injured AECs, we used MLE-12 cells, which have AECII-like characteristics, to establish an in vitro model of BLM-induced cell damage. CCK-8 assays showed that BLM at 10 µg/mL significantly reduced MLE-12 cell viability (Fig. 2A), so this concentration was used in subsequent experiments. Treatment with hucMSCs mitigated BLM-induced cytotoxicity and restored cell viability (Fig. 2B). Western blot analysis revealed a significant increase in the expression of alveolar surfactant protein SPC in the hucMSC-treated group compared to the BLM group (Fig. 2C). Immunofluorescence staining further confirmed that SPC fluorescence intensity was reduced in the BLM group, whereas the hucMSC-treated group showed a marked increase in SPC expression (Fig. 2D). These results suggest that hucMSCs effectively alleviate alveolar epithelial injury in vitro.

Next, we evaluated the therapeutic effects of hucMSCs in vivo, focusing on their ability to alleviate both epithelial injury and pulmonary fibrosis. To determine whether intravenously infused hucMSCs could localize to the injured lung, DiI-labeled hucMSCs were examined in frozen lung sections at 3 and 7 days after transplantation. DiI-positive cells were observed in the lung at both time points, supporting the ability of transplanted hucMSCs to home to the injured lung and persist during the early phase after infusion (Supplementary Fig. S5). Pulmonary function tests showed that both dynamic lung compliance (Cdyn) and forced vital capacity (FVC) were significantly reduced in BLM-induced mice, while hucMSC treatment notably improved these parameters (Fig. 2F, G). Micro-CT imaging, together with H&E and Masson’s trichrome staining, revealed that, compared to BLM-treated mice, hucMSC-treated mice exhibited thinner alveolar septa, less collagen deposition, improved alveolar architecture, and significantly reduced alveolar inflammation (Fig. 2H, I). Western blot analysis showed that the expression of fibrosis-related proteins, including collagen I, vimentin, and α-SMA, was markedly reduced in the hucMSC-treated group compared to the BLM group (Fig. 2J). Furthermore, SPC expression was significantly decreased in the lungs of BLM-treated mice, but treatment with hucMSCs restored SPC levels (Fig. 2K). Immunofluorescence staining confirmed that SPC expression was reduced in the lungs of BLM-treated mice, whereas hucMSC treatment effectively restored SPC expression (Fig. 2L). These findings indicate that hucMSCs can alleviate alveolar epithelial injury and attenuate pulmonary fibrosis in vivo.

### Proteomic analysis of BLM-injured MLE-12 cells after hucMSC treatment

**Fig. 3:**
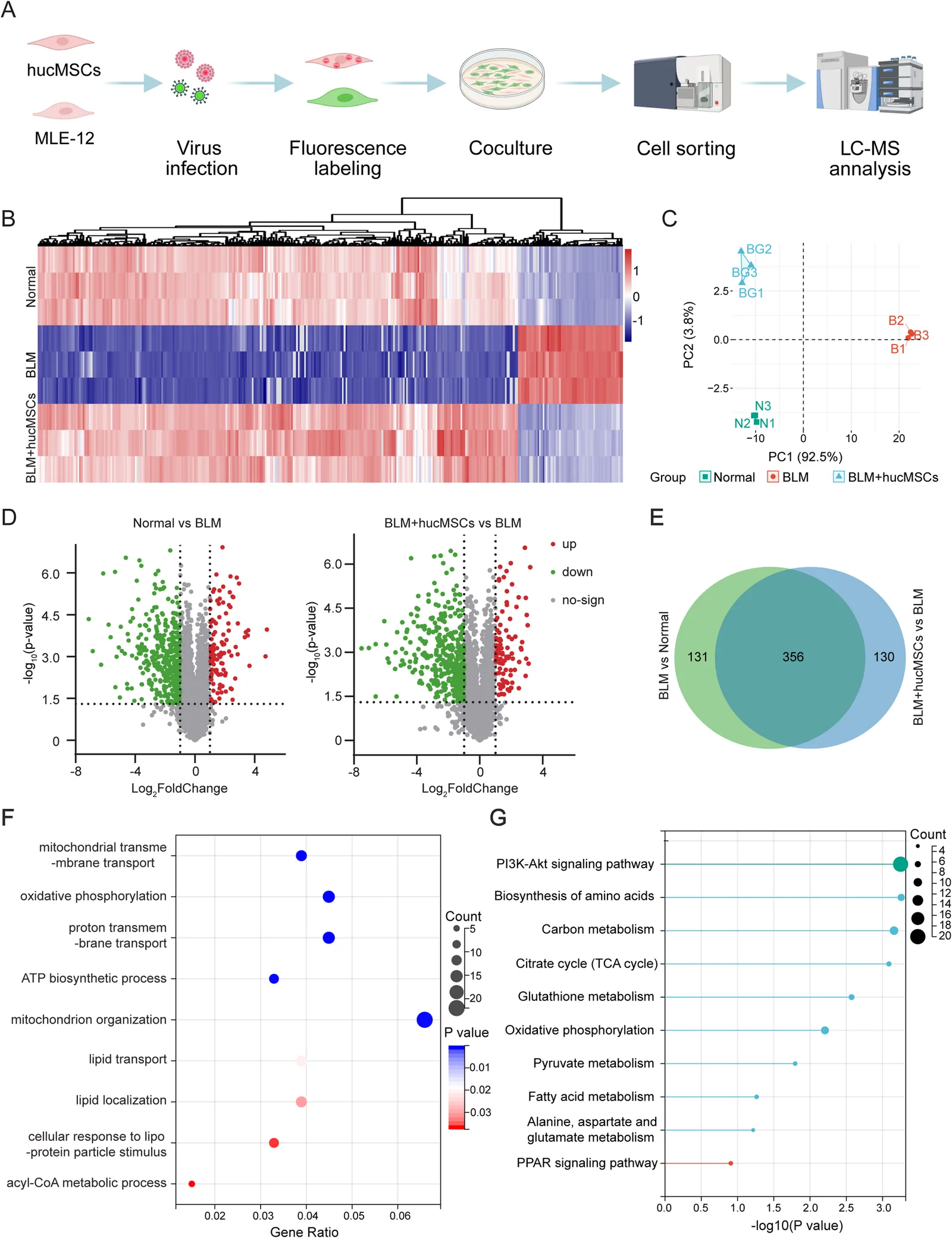
Proteomic analysis was performed on the corresponding MLE-12 cell population from each experimental group. In the BLM + hucMSCs condition, MLE-12 cells were isolated after coculture with hucMSCs prior to proteomic analysis. **A** Schematic overview of the proteomic analysis workflow. **B** Heatmap displaying DEPs across the Normal, BLM, and BLM + hucMSCs groups. Each column represents a protein, and each row corresponds to a sample from the three groups. **C** PCA showing minimal variation within each group and clear differentiation among the three groups. **D** Volcano plots of DEPs. The x-axis shows the log2 fold-change, reflecting the magnitude and direction of protein expression changes, whereas the y-axis shows the –log10 p-value, indicating statistical significance. Red and green dots represent significantly upregulated and downregulated proteins, respectively. **E** Venn diagram illustrating the overlap of DEPs between the BLM vs. Normal and BLM + hucMSCs vs. BLM comparisons. **F** GO enrichment analysis revealed distinct sets of significantly enriched terms between the BLM vs. Normal and BLM + hucMSCs vs. BLM comparisons. **G** KEGG pathway analysis showing significant enrichment of DEPs in pathways related to mitochondrial function, including the citrate cycle, oxidative phosphorylation, fatty acid metabolism, and the PPAR signaling pathway

To define the molecular changes occurring in recipient epithelial cells after hucMSC treatment, proteomic analysis was performed on the corresponding MLE-12 cell population from each experimental group rather than on mixed coculture samples. In the BLM + hucMSCs group, BLM-injured MLE-12 cells were cocultured with hucMSCs and then isolated prior to proteomic analysis (Fig. 3A). These cells were compared to those from the normal MLE-12 group and the BLM-only group. A heatmap revealed distinct patterns of differentially expressed proteins (DEPs) among the three groups (Fig. 3B). Principal component analysis (PCA) showed minimal variation within each group, with clear differences between the normal, BLM, and BLM + hucMSCs groups (Fig. 3C). Compared to the normal group, BLM treatment for 24 h resulted in 486 DEPs, of which 111 were upregulated and 375 were downregulated. When comparing the BLM + hucMSCs group to the BLM group, 487 DEPs were identified, with 393 proteins upregulated and 94 downregulated (Fig. 3D). A total of 356 proteins were differentially expressed in both comparisons. Notably, 130 proteins were unique to the BLM vs. normal group, whereas 131 proteins were unique to the BLM + hucMSCs vs. BLM group (Fig. 3E). GO and KEGG enrichment analyses were conducted to explore the biological functions and pathways of these DEPs. GO analysis revealed significant enrichment in pathways related to mitochondrial transmembrane transport, lipid transport and localization, oxidative phosphorylation, proton transmembrane transport, ATP biosynthesis, mitochondrion organization, and cellular response to lipoprotein particle stimulus, among others (Fig. 3F). These processes are all closely associated with mitochondrial function, including ATP synthesis through oxidative phosphorylation in the mitochondrial inner membrane, lipid metabolism via β-oxidation in the mitochondrial matrix, and the transport of fatty acids and metabolites across mitochondrial membranes. KEGG pathway analysis supported these findings, showing significant enrichment of DEPs in pathways related to the citrate cycle (TCA cycle), oxidative phosphorylation, fatty acid metabolism, and the PPAR signaling pathway. These pathways, all mitochondria-associated, are key regulators of energy and lipid metabolism, with the PPAR pathway modulating mitochondrial functions (Fig. 3G). Together, these results suggest that mitochondria serve as a central hub for metabolic reprogramming in the epithelial repair process mediated by hucMSCs. The treatment with hucMSCs appears to alleviate mitochondrial dysfunction in injured MLE-12 cells, reprogramming lipid metabolism and promoting recovery.

### hucMSCs transfer functional mitochondria to injured epithelial cells, contributing to epithelial recovery

Based on the mitochondrial function–related pathways identified in the proteomic analysis, we next examined whether hucMSC treatment could restore mitochondrial function in injured alveolar epithelial cells (AECs). Mitochondrial function was assessed by measuring mitochondrial membrane potential (MMP), ATP production, and reactive oxygen species (ROS) levels. Compared with the control group, BLM-treated MLE-12 cells exhibited a marked increase in ROS levels, accompanied by significant reductions in ATP production and MMP. Notably, hucMSC treatment effectively reversed these changes, as evidenced by decreased ROS levels and restoration of ATP production and MMP (Supplementary Fig. S1). These results indicate that hucMSCs alleviate mitochondrial dysfunction in injured epithelial cells.

**Fig. 4:**
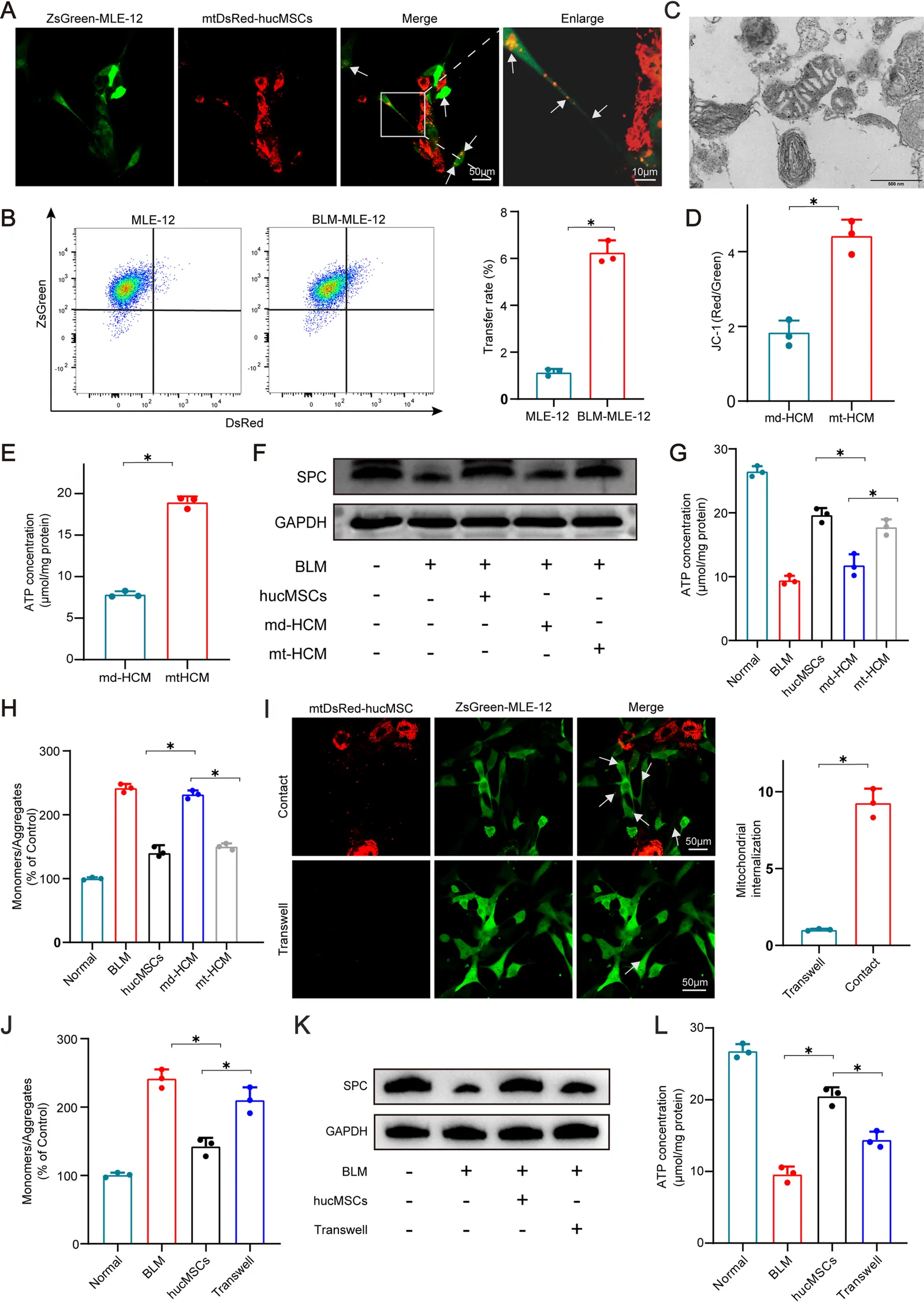
hucMSCs transfer functional mitochondria to injured epithelial cells, contributing to epithelial recovery. **A** Confocal microscopy images showing the internalization of hucMSC-derived mitochondria (red) into ZsGreen-expressing MLE-12 cells in the coculture system. **B** Flow cytometric analysis demonstrated that BLM-injured MLE-12 cells received significantly more mitochondria from hucMSCs than normal MLE-12 cells. **C** Transmission electron microscopy (TEM) images showing mitochondria with intact double-membrane structures and well-preserved cristae in hucMSC-conditioned medium. **D**, **E** JC-1 and ATP assays showed that mitochondria-containing hucMSC-conditioned medium (mt-HCM) exhibited significantly higher mitochondrial membrane potential (MMP) and ATP levels than mitochondria-depleted conditioned medium (md-HCM). **F**–**H** Compared with md-HCM, mt-HCM more effectively restored epithelial function in BLM-injured MLE-12 cells, as evidenced by increased SPC expression, enhanced ATP production, and improved MMP. **I** Immunofluorescence analysis showed that mitochondrial internalization into MLE-12 cells was markedly reduced under Transwell coculture conditions compared with direct coculture. **J** JC-1 staining showed that the recovery of MMP in BLM-injured MLE-12 cells was significantly attenuated under Transwell coculture conditions compared with direct coculture. **K** Western blot analysis of SPC expression in MLE-12 cells under different conditions. hucMSC treatment increased SPC expression in BLM-injured cells, whereas this effect was attenuated under Transwell coculture conditions. GAPDH was used as a loading control. **L** ATP levels in MLE-12 cells under direct coculture or Transwell conditions. Compared with direct coculture, Transwell significantly reduced the ability of hucMSCs to restore ATP production in BLM-injured cells. Data are presented as mean ± SD, *n* = 3, *P<0.05

We next investigated whether this functional recovery was associated with mitochondrial transfer from hucMSCs to epithelial cells. To directly visualize mitochondrial transfer, a fluorescence-based coculture system was established by labeling donor hucMSC mitochondria with mitochondria-targeted DsRed and recipient MLE-12 cells with cytoplasmic ZsGreen. Confocal microscopy demonstrated that mitochondria derived from hucMSCs were internalized into MLE-12 cells (Fig. 4A).

To quantify mitochondrial transfer efficiency, flow cytometric analysis was performed. Compared with normal MLE-12 cells, BLM-injured MLE-12 cells exhibited a significantly higher proportion of mitochondria-positive cells following coculture with hucMSCs (Fig. 4B), indicating that injured epithelial cells possess an enhanced capacity to receive mitochondria.

Given that hucMSCs may exert therapeutic effects through both mitochondrial transfer and paracrine mechanisms, we next examined whether functional mitochondria are released into the extracellular environment. Transmission electron microscopy (TEM) revealed the presence of mitochondria with intact double membranes and well-preserved cristae in extracellular fractions derived from hucMSC-conditioned medium (Fig. 4C). To further evaluate their functional status, we compared mitochondria-containing conditioned medium (mt-HCM) with mitochondria-depleted conditioned medium (md-HCM). mt-HCM exhibited significantly higher mitochondrial membrane potential and ATP levels than md-HCM (Fig. 4D, E), indicating that hucMSCs release metabolically active mitochondria.

To determine the functional contribution of secreted mitochondria, injured MLE-12 cells were treated with mt-HCM or md-HCM. While md-HCM exerted a modest protective effect, mt-HCM more effectively restored epithelial function, as evidenced by increased SPC expression, enhanced ATP production, and improved MMP (Fig. 4F–H). These findings indicate that both mitochondrial and non-mitochondrial paracrine factors contribute to epithelial recovery, with secreted mitochondria representing a major functional component of the hucMSC secretome.

To further assess whether mitochondrial transfer is functionally required for hucMSC-mediated epithelial protection, we used a Transwell coculture system, as previous studies have shown that, compared with direct-contact coculture, Transwell separation significantly suppresses intercellular mitochondrial transfer. This approach allowed us to evaluate the protective effects of hucMSCs under conditions of reduced mitochondrial transfer. Immunofluorescence analysis confirmed that mitochondrial internalization into MLE-12 cells was markedly reduced under Transwell conditions compared with direct coculture (Fig. 4I). Consistently, the ability of hucMSCs to restore mitochondrial function was significantly attenuated, as reflected by reduced recovery of MMP (Fig. 4J), as well as diminished SPC expression and ATP production (Fig. 4K-L).

### Proteomic analysis of hucMSCs reveals molecular changes associated with mitochondrial transfer

**Fig. 5:**
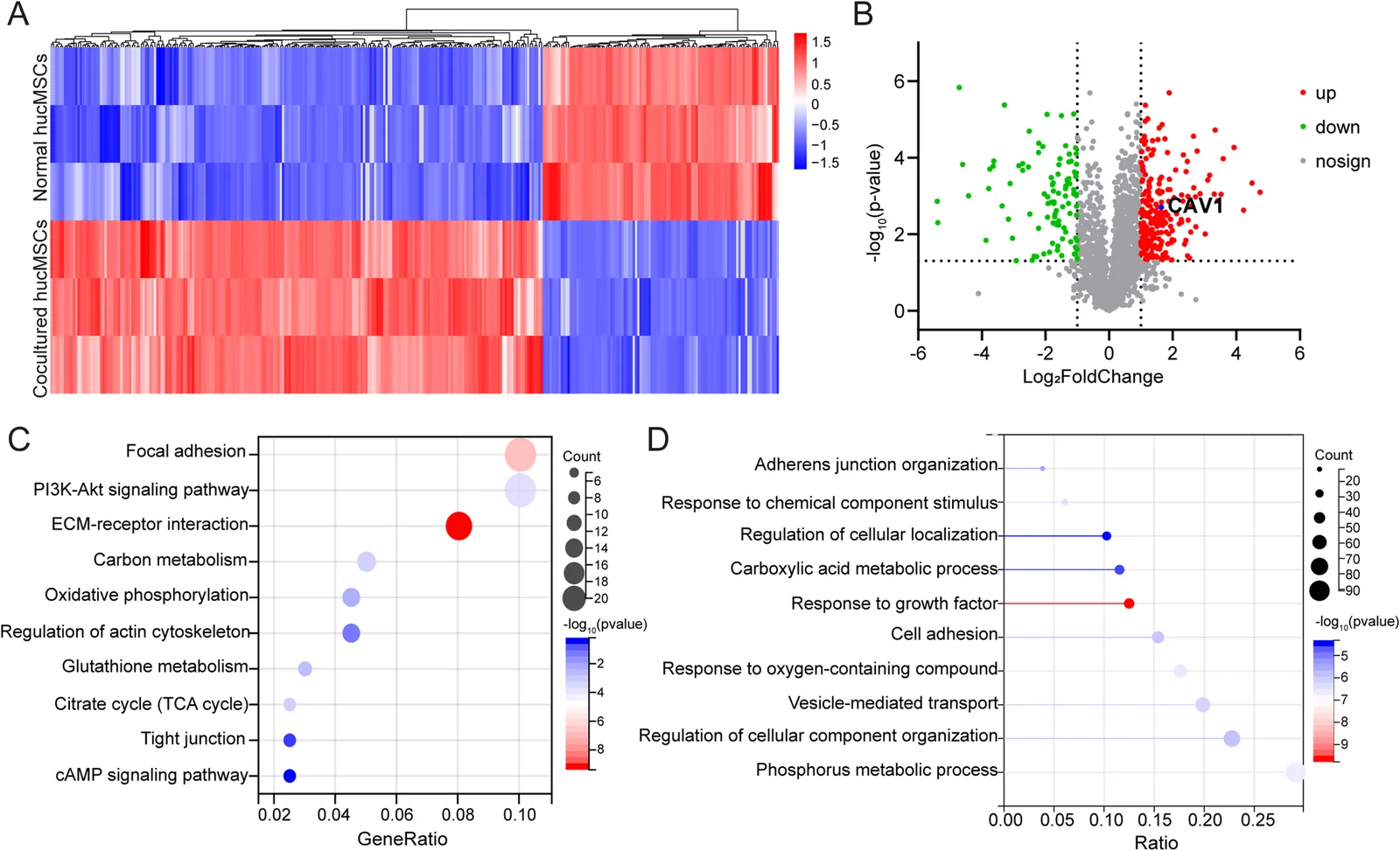
Proteomic analysis of hucMSCs after coculture with BLM-treated MLE-12 cells. **A** Heatmap showing DEPs between normal hucMSCs and cocultured hucMSCs. **B** Volcano plot highlighting significant differences in protein expression between cocultured and normal hucMSCs. **C** KEGG pathway analysis showed that DEPs in cocultured hucMSCs were significantly enriched in pathways related to oxidative phosphorylation, regulation of the actin cytoskeleton, and tight junctions. **D** GO enrichment analysis revealed that DEPs in cocultured hucMSCs were predominantly associated with vesicle-mediated transport, regulation of cellular localization, response to chemical component stimulus andresponse to oxygen-containing compound compared to normal hucMSCs

To further characterize the molecular alterations in donor hucMSCs during coculture with injured epithelial cells, we performed proteomic analysis on hucMSCs after coculture with BLM-injured MLE-12 cells. A heatmap revealed distinct protein expression profiles between normal hucMSCs and cocultured hucMSCs (Fig. 5A). Differentially expressed proteins (DEPs) were defined using a threshold of *p* < 0.05 and fold change > 2 or < 0.5. In total, 324 DEPs were identified in cocultured hucMSCs compared with normal controls, including 219 upregulated and 105 downregulated proteins (Fig. 5B). KEGG pathway enrichment analysis showed that these DEPs were significantly enriched in pathways related to oxidative phosphorylation, regulation of the actin cytoskeleton, and tight junctions (Fig. 5C), suggesting alterations in mitochondrial metabolism, cytoskeletal organization, and intercellular interaction in cocultured hucMSCs. GO enrichment analysis further revealed enrichment in biological processes such as vesicle-mediated transport, regulation of cellular localization, cell adhesion, and responses to chemical and oxidative stimuli (Fig. 5D), indicating that hucMSCs undergo adaptive molecular remodeling in response to the injured epithelial microenvironment. Notably, CAV1 was identified as a significantly upregulated protein in cocultured hucMSCs and was highlighted in the volcano plot (Fig. 5B). Western blot analysis further confirmed the increased endogenous expression of CAV1 in cocultured hucMSCs (Supplementary Fig. S4). Importantly, several enriched pathways, including actin cytoskeleton regulation, vesicle-mediated transport, and cell adhesion, are closely related to membrane dynamics and intracellular trafficking, processes in which CAV1 has been reported to play regulatory roles. Collectively, these proteomic data indicate that cocultured hucMSCs undergo coordinated molecular remodeling involving mitochondrial metabolism, cytoskeletal organization, and vesicle-associated trafficking. While these findings do not directly demonstrate mitochondrial transfer, they are consistent with an enhanced donor-cell state that may facilitate intercellular mitochondrial trafficking to injured epithelial cells.

### CAV1 promotes hucMSC mitochondrial transfer to facilitate the recovery of mitochondrial function in injured epithelial cells

**Fig. 6:**
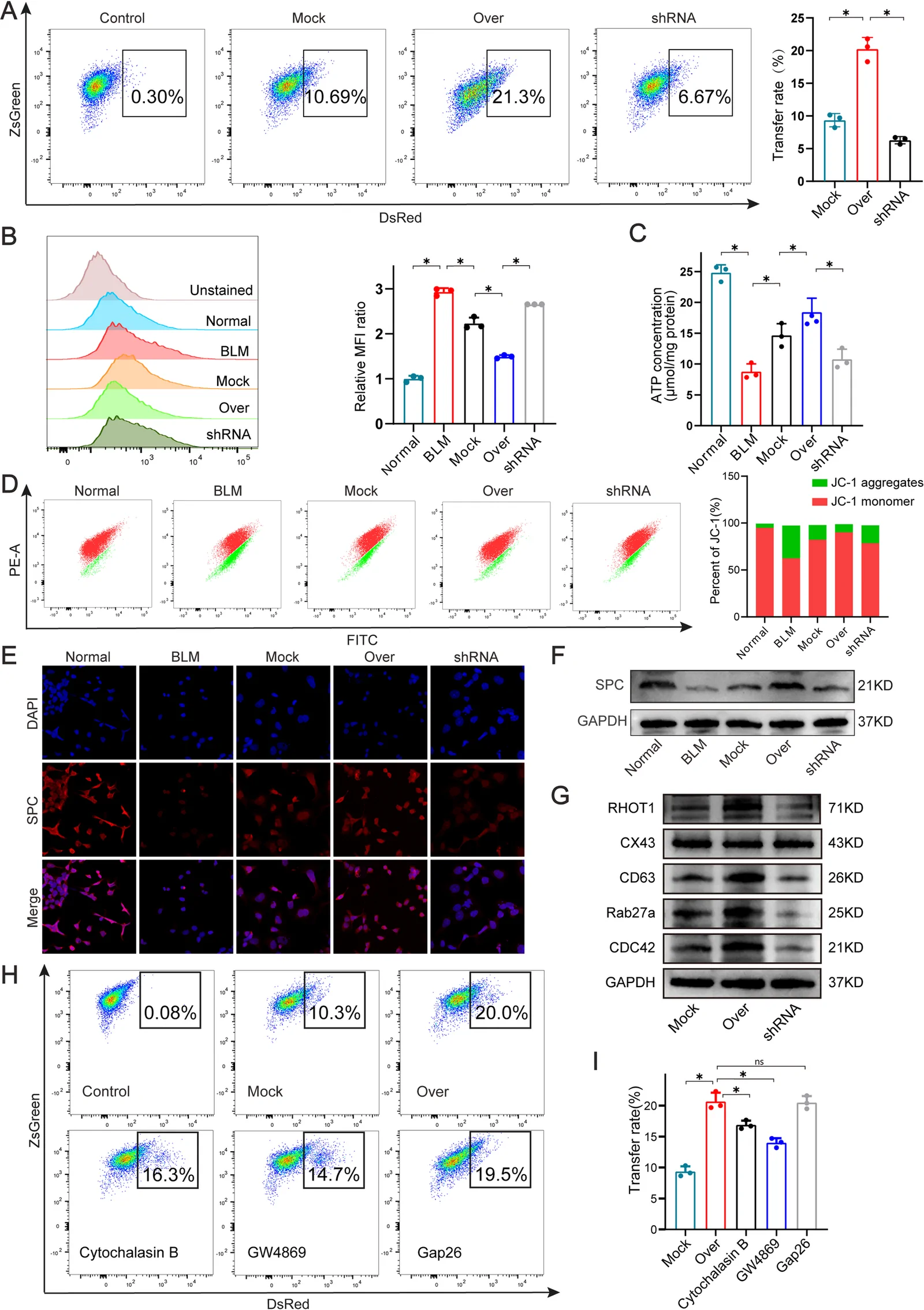
CAV1 promotes hucMSC mitochondrial transfer to facilitate the recovery of mitochondrial function in injured epithelial cells. **A** Mitochondrial transfer rates from hucMSCs in the Mock, Over, and shRNA groups to BLM-treated MLE-12 cells. **B** ROS levels in MLE-12 cells cocultured with hucMSCs from the Mock, Over, and shRNA groups. **C** ATP quantification assay showing significantly restored ATP levels in the Over and Mock groups compared to the BLM group, with ATP levels remaining low in the shRNA group. **D** JC-1 staining assay measuring MMP. The Over group showed a markedly higher red/green fluorescence ratio compared to the BLM, Mock, and shRNA groups. **E** Immunofluorescence staining of SPC expression in MLE-12 cells. CAV1 overexpression in hucMSCs restored SPC expression compared to the BLM and other groups. **F** Western blot analysis of SPC protein expression in MLE-12 cells under different treatment conditions. Coculture with hucMSCs restored SPC expression after BLM injury, which was further enhanced by CAV1 overexpression and attenuated by CAV1 knockdown. **G** Western blot analysis of mitochondrial transfer-related proteins in hucMSCs after CAV1 manipulation. hucMSCs were recovered from the coculture system by cell sorting for protein analysis. RHOT1, CDC42, CD63, and Rab27a were increased in the Over group and showed a downward trend in the shRNA group, whereas CX43 did not show a corresponding change. **H** Representative flow cytometry plots showing mitochondrial transfer from hucMSCs to MLE-12 cells under different conditions. CAV1 overexpression significantly increased mitochondrial transfer compared with the Mock group. Treatment with Cytochalasin B or GW4869 reduced the transfer efficiency, whereas Gap26 had no obvious effect. **I** Quantitative analysis of mitochondrial transfer efficiency based on flow cytometry. Data are presented as mean ± SD, *n* = 3, *P < 0.05

Having identified CAV1 as an upregulated candidate protein in cocultured hucMSCs by proteomic analysis, and further confirming its increased endogenous expression by western blot, we next examined whether CAV1 regulates mitochondrial transfer from hucMSCs to injured epithelial cells. Notably, the enriched pathways in cocultured hucMSCs, including actin cytoskeleton regulation, vesicle-mediated transport, and cell adhesion, are closely related to membrane dynamics and intracellular trafficking, processes in which CAV1 has recognized regulatory functions. We therefore hypothesized that CAV1 may facilitate mitochondrial transfer in the coculture system. To test this, we transduced hucMSCs with either a Mock vector, a CAV1-overexpression vector, or a CAV1-knockdown vector, which we referred to as the Mock, Over, and shRNA groups, respectively. Mitochondrial transfer from hucMSCs (in each of these groups) to BLM-treated MLE-12 cells was assessed by flow cytometry, and the transfer rates were compared. hucMSCs overexpressing CAV1 showed a significant increase in mitochondrial transfer to MLE-12 cells, whereas knockdown of CAV1 significantly reduced the transfer rate (Fig. 6A). These findings suggest that CAV1 overexpression enhances mitochondrial transfer from hucMSCs to injured epithelial cells. Further analysis revealed that ROS levels were significantly reduced in the Over group, but were elevated in the shRNA group, although ROS levels in all three groups remained lower than those in the BLM group (Fig. 6B). Quantitative ATP analysis showed that ATP levels were significantly higher in both the Mock and Over groups compared to the BLM or shRNA groups (Fig. 6C). MMP was assessed using the fluorescent probe JC-1, and the Over group exhibited a significantly higher MMP compared to the BLM, Mock, and shRNA groups (Fig. 6D). Both immunofluorescence staining and western blot (Fig. 6E, F) analysis further confirmed that SPC expression was significantly higher in the Over group compared to the BLM group and the other two groups. Together, these results indicate that CAV1 overexpression in hucMSCs promotes mitochondrial transfer to injured AECs, which helps restore MMP and ATP production, reduces oxidative stress, and ultimately alleviates epithelial cell injury.

To further investigate how CAV1 enhances mitochondrial transfer, we examined proteins related to cytoskeletal remodeling, vesicle trafficking, and gap-junction communication in hucMSCs sorted from the hucMSC–MLE-12 coculture system after CAV1 manipulation. Western blot analysis showed that RHOT1, CD63, Rab27a, and CDC42 were increased in the CAV1-overexpression group and showed a downward trend after CAV1 knockdown. In contrast, CX43 did not exhibit a corresponding change pattern with CAV1 manipulation (Fig. 6G). To assess the functional relevance of these pathways, CAV1-overexpressing hucMSCs were pretreated with Cytochalasin B, GW4869, or Gap26, which were selected based on previous reports as pharmacological inhibitors of mitochondrial transfer-related pathways, before coculture with BLM-injured MLE-12 cells [23–25]. Flow cytometric analysis demonstrated that the CAV1-induced increase in mitochondrial transfer was significantly attenuated by Cytochalasin B and GW4869, whereas Gap26 had no obvious effect (Fig. 6H, I). These findings suggest that CAV1-facilitated mitochondrial transfer is more closely associated with cytoskeleton-dependent processes and extracellular vesicle-related pathways than with CX43-mediated gap-junction communication.

### Overexpression of CAV1 in hucMSCs alleviates alveolar epithelial injury and attenuates pulmonary fibrosis in vivo

**Fig. 7:**
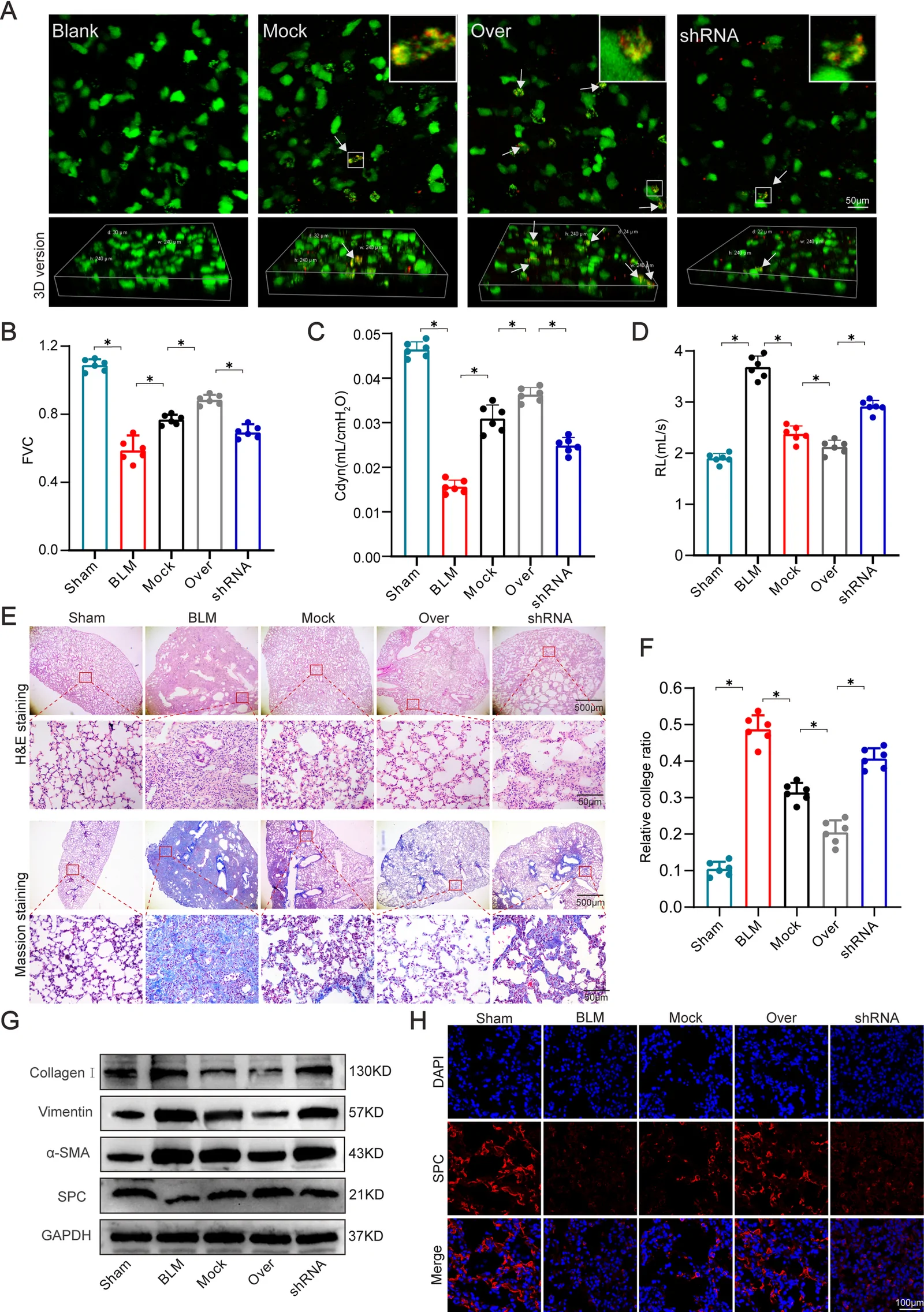
CAV1 overexpression in hucMSCs alleviated alveolar epithelial injury to attenuate pulmonary fibrosis in vivo. **A** hucMSCs were labeled with DsRed (red) to track donor cell mitochondria, and AECs were labeled with ZsGreen (green). Fluorescence imaging revealed enhanced mitochondrial transfer in the CAV1 overexpression group and reduced transfer in the CAV1 knockdown group compared to the Mock control. **B** Flow-volume curves showing improved lung function in the Over group compared to the BLM, Mock, and shRNA groups. **C** Dynamic compliance (Cdyn) analysis demonstrated that compliance was restored in the Over group but remained low in the shRNA group. **D** RL measurements showed decreased resistance in the Over group compared to the other groups. **E** Representative images from H&E and Masson’s trichrome staining showing alveolar structure and collagen deposition. **F** Quantification of collagen area fraction from Masson’s staining, confirming reduced fibrosis in the Over group. **G** Western blot analysis of fibrosis-related proteins (collagen I, vimentin, and α-SMA), with GAPDH as the loading control. Protein levels were markedly reduced in the Over group compared to the BLM, Mock, and shRNA groups. **H** Immunofluorescence staining of SPC showing recovery of AECIIs in the Over group, with stronger fluorescence intensity compared to the other groups. Data are presented as mean ± SD, *n* = 6, *P < 0.05

To investigate the role of CAV1 in hucMSC-mediated protection against pulmonary fibrosis, a BLM-induced mouse model was established and treated the mice with hucMSCs carrying lentiviral vectors for CAV1 overexpression (Over), CAV1 knockdown (shRNA), or an empty control (Mock). In vivo lung imaging was performed to directly visualize mitochondrial transfer from hucMSCs in the fibrotic lung. Fluorescence imaging showed that hucMSCs with CAV1 overexpression exhibited significantly enhanced mitochondrial transfer to AECs, whereas CAV1 knockdown hucMSCs displayed notably reduced mitochondrial transfer (Fig. 7A). Flow-volume curves revealed significant impairment in lung function in the BLM group compared to the sham group, as evidenced by reduced peak flow and volume (Fig. 7B). CAV1 overexpression in hucMSCs (Over group) significantly improved lung function, whereas the shRNA group showed further deterioration compared to the Mock group. Dynamic compliance (Cdyn) analysis showed that lung compliance was notably decreased in the BLM group. This reduction was partially reversed in the Mock group and more significantly restored in the Over group, while compliance remained lowest in the shRNA group (Fig. 7C). Similarly, lung resistance (RL) was significantly increased in the BLM group, moderately reduced in the Mock group, and most significantly improved in the Over group, whereas the shRNA group maintained high resistance (Fig. 7D). Histological analysis through H&E and Masson’s staining revealed severe alveolar structural damage and extensive collagen deposition in the BLM group. These pathological changes were alleviated in the Mock group and substantially improved in the Over group, while fibrosis progression remained apparent in the shRNA group (Fig. 7E). Quantitative analysis of collagen area fraction confirmed that collagen deposition was significantly lower in the Over group compared to the BLM, Mock, and shRNA groups (Fig. 7F). Western blot analysis showed that the expression of fibrosis-related proteins, including collagen I, vimentin, and α-SMA, was markedly elevated in the BLM group, reduced in the Mock group, and most significantly downregulated in the Over group. In contrast, the shRNA group showed persistently high expression of these fibrotic markers (Fig. 7G). Immunofluorescence staining also revealed that SPC expression, a marker for alveolar type II epithelial cells (AECIIs), was significantly diminished in the BLM group, partially recovered in the Mock group, and most strongly preserved in the Over group, whereas the shRNA group showed little improvement (Fig. 7H). Overall, these findings suggest that CAV1 overexpression in hucMSCs helps to alleviate alveolar epithelial injury and significantly attenuate pulmonary fibrosis in vivo.

### hucMSCs-derived mitochondrial transfer reprograms lipid metabolism in BLM-injured MLE-12 cells

**Fig. 8:**
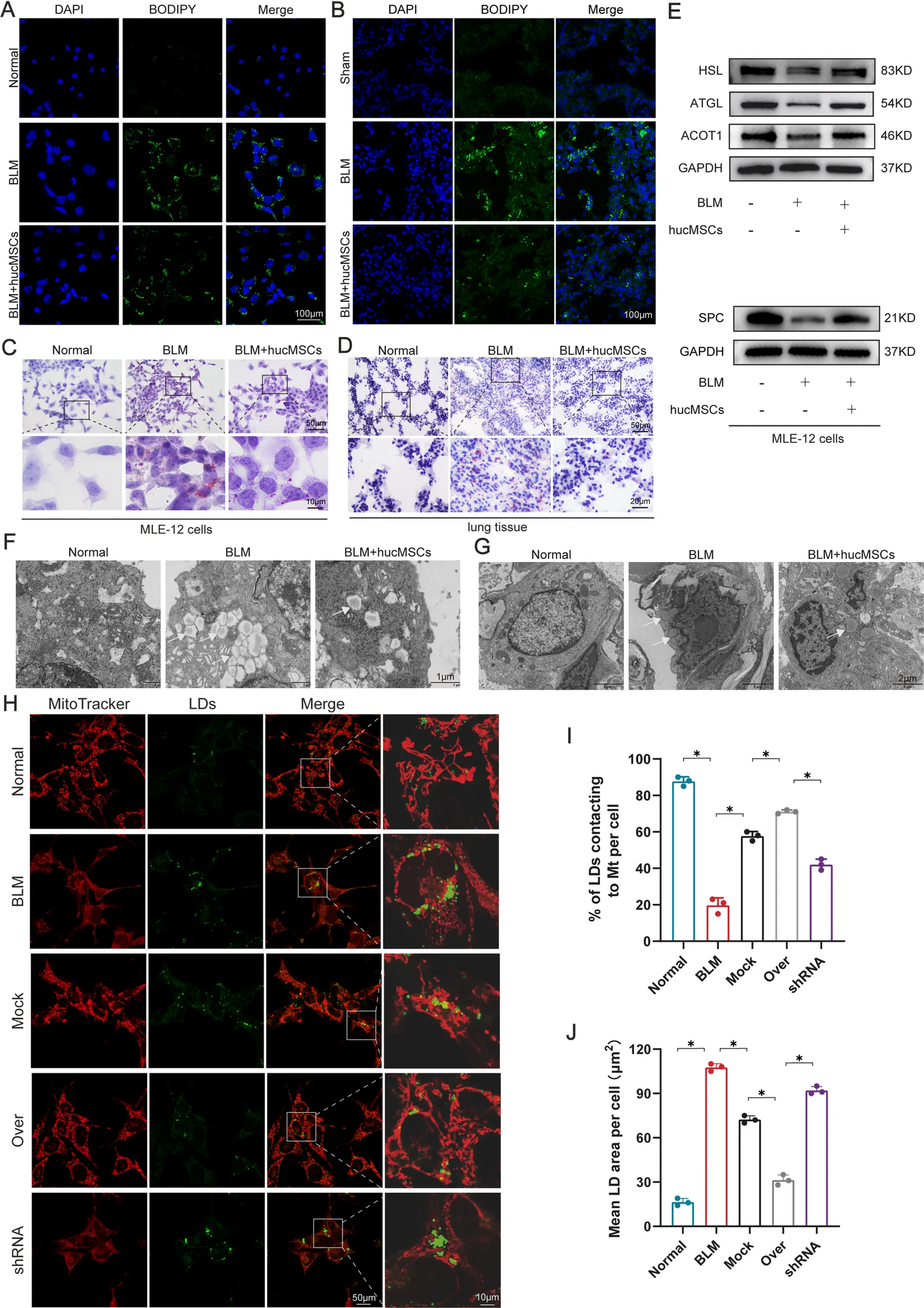
Mitochondrial transfer from hucMSCs promotes lipid metabolic reprogramming in injured AECs. **A** LD accumulation was significantly reduced in the BLM + hucMSCs group compared to the BLM group in MLE-12 cells. **B** In lung tissues, AECs from the BLM + hucMSCs group exhibited a marked decrease in LDs compared to the BLM group. **C** Oil Red O staining in MLE-12 cells revealed significant LD accumulation in the BLM group, which was notably reduced in number and size following hucMSC treatment. **D** In lung tissues, AECs from the BLM group showed intense Oil Red O-positive LDs, while those in the BLM + hucMSCs group exhibited markedly fewer and smaller LDs. **E** Western blot analysis showed increased expression of lipolysis-associated proteins (HSL, ATGL, ACOT1) in the BLM + hucMSCs group compared to the BLM group. **F**, **G** TEM analysis of MLE-12 cells and lung tissues revealed increased LD accumulation in the BLM group, whereas the number of LDs was markedly reduced in the BLM + hucMSCs group. **H** MLE-12 cells from the Normal, BLM, Mock, Over, and shRNA groups were stained with BODIPY 493/503 (green) to label LDs and MitoTracker Red (red) to label mitochondria. **I** Quantification of the percentage of LDs in contact with mitochondria (Mt) per cell in each group. MLC was significantly reduced in the BLM group compared to the normal group, but hucMSC treatment (Mock) restored MLC. CAV1 overexpression (Over) further enhanced MLC, whereas CAV1 knockdown (shRNA) led to reduced MLC. **J** Quantitative analysis of the mean LD area per cell. BLM treatment significantly increased LD size, which was alleviated by hucMSC treatment. CAV1 overexpression (Over) further decreased LD size, whereas CAV1 knockdown (shRNA) led to an increase. Data are presented as mean ± SD, *n* = 3, *P < 0.05

To further validate the proteomic findings and investigate how hucMSC-mediated mitochondrial transfer affects lipid metabolism, we assessed LD accumulation and the expression of key proteins related to lipid metabolism. Using BODIPY 493/503 staining, a fluorescent dye that specifically labels neutral lipids and LDs, we observed an increase in both the number and size of LDs in MLE-12 cells from the BLM group. In lung tissue, BODIPY staining also revealed marked accumulation of neutral lipids within the alveolar regions of BLM-treated mice. In contrast, MLE-12 cells from the BLM + hucMSCs group and corresponding lung tissue showed a significant reduction in both the number and size of LDs (Fig. 8A, B). Oil Red O staining further confirmed these observations (Fig. 8C, D). Next, we performed Western blotting to examine how mitochondrial transfer from hucMSCs affects key regulators of lipid metabolism in injured AECs. We focused on proteins such as hormone-sensitive lipase (HSL), adipose triglyceride lipase (ATGL), and acyl-CoA thioesterase 1 (ACOT1), which are involved in lipid hydrolysis and fatty acid mobilization. Compared to the BLM group, the BLM + hucMSCs group showed increased expression of HSL, ATGL, and ACOT1, suggesting enhanced lipid breakdown and fatty acid release (Fig. 8E). These results imply that mitochondrial transfer from hucMSCs promotes lipid catabolic activity in injured AECs. TEM images revealed significant LD accumulation in MLE-12 cells and lung tissues from the BLM group. Remarkably, hucMSC treatment reduced both the number and size of LDs compared to the BLM group, suggesting that hucMSCs help mitigate BLM-induced lipid deposition and restore lipid metabolic balance (Fig. 8F, G).

To further investigate how hucMSC-mediated mitochondrial transfer alleviates lipid accumulation, we analyzed the interaction between mitochondria and LDs by examining mitochondria–lipid droplet contact (MLC). Mitochondria (red) and LDs (green) in MLE-12 cells were fluorescently labeled to observe MLC. Fluorescence imaging revealed that in the normal group, cells had few LDs, with over 80% of them in close contact with mitochondria. In contrast, BLM-treated cells showed a marked increase in LD area and a decrease in MLC. These alterations were alleviated following hucMSC treatment. Interestingly, CAV1 overexpression in hucMSCs significantly decreased the average LD area and restored MLC, whereas CAV1 knockdown resulted in reduced MLC in MLE-12 cells (Fig. 8H-J). Together, these findings suggest that mitochondrial transfer from hucMSCs restores the physical interaction between mitochondria and LDs, promoting lipid utilization in injured AECs. These results also demonstrate that CAV1 enhances hucMSC-mediated mitochondrial transfer, reinforcing mitochondria–LD interactions, which ultimately restores lipid metabolic homeostasis in epithelial cells.

## Discussion

hucMSC-based therapy offers significant potential for treating pulmonary fibrosis, thanks to its strong anti-inflammatory and regenerative properties [26–28]. However, the exact mechanisms behind its therapeutic effects are not fully understood, which limits its broader clinical application [29–31]. In this study, we demonstrated that hucMSCs alleviate BLM-induced pulmonary fibrosis by transferring functional mitochondria to injured AECs. This mitochondrial transfer helped restore mitochondrial integrity, reduce oxidative stress, and enhance epithelial repair, both in vitro and in vivo. Mechanistically, we found that CAV1 played a crucial role in mediating the transfer of mitochondria from hucMSCs to epithelial cells. CAV1-dependent mitochondrial delivery promoted the interaction between mitochondria and LDs in the recipient epithelial cells, enhancing fatty acid β-oxidation and correcting lipid metabolic dysregulation. These findings introduce a novel CAV1-regulated mitochondrial transfer mechanism through which hucMSCs restore epithelial lipid homeostasis and slow the progression of pulmonary fibrosis.

Recent studies have highlighted the multifaceted role of CAV1 in maintaining mitochondrial homeostasis, regulating membrane dynamics, and facilitating cellular communication [32–34]. As a cholesterol-binding scaffolding protein and a major structural component of caveolae, CAV1 is closely linked with membrane lipid composition and fluidity [35, 36]. Disruptions in the cholesterol/CAV1/caveolae system can alter the stiffness of MSC membranes, their substrate adhesion, and integrin expression, which in turn affects their mechanosensing abilities and intercellular communication [37]. Additionally, CAV1 has been shown to regulate mitochondrial quality control by inhibiting excessive mitochondrial fission and promoting mitophagy, positioning it as a key player in mitochondrial dynamics and oxidative stress regulation [38]. Moreover, priming MSCs with cholesterol-regulating agents such as tauroursodeoxycholic acid (TUDCA) has been found to enhance CAV1 expression, boost exocytosis, and double the yield of extracellular vesicles, highlighting CAV1’s role in vesicular transport and intercellular material transfer via mitochondria or vesicles [39]. Our study further supports the idea that CAV1 may facilitate mitochondrial transfer from hucMSCs by optimizing membrane microdomain organization, improving mitochondrial trafficking, and enhancing extracellular vesicle biogenesis and release. We showed that CAV1 overexpression increased mitochondrial transfer from hucMSCs to injured epithelial cells and significantly improved their therapeutic efficacy in treating pulmonary fibrosis.

Lipid metabolism is critical to normal lung physiology, as the lungs are lipid-rich organs with high rates of lipid turnover [40–42]. Increasing evidence suggests that lipid metabolism dysregulation is a key factor contributing to IPF [43, 44]. In fibrotic lungs, mitochondrial dysfunction and impaired lipid handling create a vicious cycle of oxidative stress and epithelial damage [45–47]. For instance, inhibiting Drp1 prevents mitochondrial fission in fibroblasts and reprograms lipid metabolism through the ROS/HIF-1α signaling axis, which in turn suppresses fibroblast activation and attenuates fibrosis progression [48]. Similarly, the expression of 3-hydroxy-3-methylglutaryl-CoA synthase 2 (HMGCS2) is significantly reduced in AECIIs, and its restoration alleviates lung fibrosis by promoting lipid degradation through upregulation of CPT1A and CPT2 via interaction with PPARα [49]. On the other hand, ACSS3 impairs fatty acid oxidation through CPT1A deficiency, which leads to enhanced glycolysis, increased ROS production, defective mitophagy, and epithelial apoptosis—features that characterize metabolic reprogramming in IPF [50]. These findings highlight the close connection between mitochondrial dysfunction and lipid metabolic disturbances in the development of pulmonary fibrosis. In this context, restoring mitochondrial function presents a promising strategy to correct lipid metabolic imbalances. Our study demonstrates that hucMSC-mediated mitochondrial transfer effectively alleviates epithelial lipid metabolic dysregulation by restoring mitochondrial homeostasis, thereby reducing epithelial injury and mitigating fibrosis progression.

In summary, our study demonstrates that CAV1 augments hucMSC-mediated mitochondrial transfer to improve mitochondrial function and lipid metabolic homeostasis, thereby mitigating epithelial injury; nevertheless, several limitations remain. Although our supplementary experiments provide preliminary evidence that CAV1-facilitated mitochondrial transfer is associated with cytoskeleton-dependent and extracellular vesicle–related pathways, the exact transfer mechanism remains incompletely understood. Further studies will be required to determine whether CAV1 mainly regulates TNT formation, vesicle-mediated transport, or multiple parallel routes. It should also be noted that no immunosuppressive pretreatment was used in this xenogeneic transplantation model, and potential host immune responses cannot be completely excluded. Moreover, tracking of labeled hucMSCs indicated transient pulmonary retention after infusion rather than durable long-term persistence, suggesting that the observed therapeutic effects were more likely mediated by early post-infusion events.

## Conclusion

Overexpression of CAV1 enhances the transfer of mitochondria from hucMSCs to injured epithelial cells, restoring mitochondrial function and promoting mitochondria–LD coupling. This CAV1-dependent mitochondrial transfer reprograms epithelial lipid metabolism, thereby alleviating pulmonary fibrosis. These findings provide a promising strategy to enhance the therapeutic efficacy of stem cell–based treatments for fibrotic lung diseases.

## Supplementary Information

Below is the link to the electronic supplementary material.

Supplementary Material 1.

Supplementary Material 2.

Supplementary Material 3.

Supplementary Material 4.

Supplementary Material 5.

Supplementary Material 6.

## Data Availability

The proteomics datasets generated in this study have been deposited in the iProX repository under accession number PXD072122. Additional information is available from the corresponding author upon reasonable request.

## Abbreviations

IPF: Idiopathic pulmonary fibrosis
MSCs: Mesenchymal stem cells
CAV1: Caveolin-1
hucMSCs: Human umbilical-cord-derived MSCs
AECs: Alveolar epithelial cells
ECM: Rxcessive extracellular matrix
ROS: Reactive oxygen species
TNTs: Tunneling nanotubes
Mt-HCM: hucMSC-conditioned medium
Md-HCM: Mitochondria-depleted medium
MMP: Measurement of mitochondrial membrane potential
KEGG: Kyoto Encyclopedia of Genes and Genomes
GO: Gene Ontology
DEPs: Differentially expressed proteins
EVs: Extracellular vesicles
LD: Lipid droplet
Cdyn: Dynamic compliance
RL: Lung resistance
MLC: Mitochondria–lipid droplet contact

## References

[1] Khor YH, Ng Y, Barnes H, Goh NSL, McDonald CF, Holland AE. Prognosis of idiopathic pulmonary fibrosis without anti-fibrotic therapy: a systematic review. Eur Respir Rev. 2020;29:190158. 32759374 10.1183/16000617.0158-2019 PMC9488716

[2] Chen S, Wang Y, Zhang J, Liu B, Liu W, Cao G, Li R, Li H, Zhai N, Song X, et al. YTHDC1 phase separation drives the nuclear export of m6A-modified lnc NONMMUT062668.2 through the transport complex SRSF3–ALYREF–XPO5 to aggravate pulmonary fibrosis. Cell Death Dis. 2025;16:279. 40221424 10.1038/s41419-025-07608-x PMC11993731

[3] Yue D, Zhang Q, Zhang J, Liu W, Chen L, Wang M, Li R, Qin S, Song X, Ji Y. Diesel exhaust PM₂.₅ greatly deteriorates fibrosis process in pre-existing pulmonary fibrosis via ferroptosis. Environ Int. 2023;171:107706. 36565570 10.1016/j.envint.2022.107706

[4] Confalonieri P, Volpe MC, Jacob J, Maiocchi S, Salton F, Ruaro B, Confalonieri M, Braga L. Regeneration or repair? The role of alveolar epithelial cells in the pathogenesis of idiopathic pulmonary fibrosis (IPF). Cells 2022; 11:2095. 10.3390/cells11132095 PMC9266271 35805179

[5] DeLeon-Pennell KY, Barker TH, Lindsey ML. Fibroblasts: The arbiters of extracellular matrix remodeling. Matrix Biol. 2020;91–92:1–7. 32504772 10.1016/j.matbio.2020.05.006 PMC7434687

[6] Carmona-Pírez J, Salsoso R, Charpentier E, Olmedo C, Medrano FJ, Román L, de la Horra C, de Armas Y, Calderón EJ, Friaza V. Proteomic approach to study the effect of Pneumocystis jirovecii colonization in idiopathic pulmonary fibrosis. J Fungi. 2025;11:102. 10.3390/jof11020102 PMC11857022 39997396

[7] Qiu C, Zhao Z, Xu C, Yuan R, Ha Y, Tu Q, Zhang H, Mu Z, Xin Q, Tian Y, et al. Nebulized milk exosomes loaded with siTGF-β1 ameliorate pulmonary fibrosis by inhibiting EMT pathway and enhancing collagen permeability. J Nanobiotechnol. 2024;22:434. 10.1186/s12951-024-02721-z PMC11267965 39044233

[8] Suezawa T, Kanagaki S, Moriguchi K, Masui A, Nakao K, Toyomoto M, Tamai K, Mikawa R, Hirai T, Murakami K, et al. Disease modeling of pulmonary fibrosis using human pluripotent stem cell-derived alveolar organoids. Stem Cell Rep. 2021;16:2973–87. 10.1016/j.stemcr.2021.10.015 PMC8693665 34798066

[9] Hu W, Wang Y, Yang H, Zhang L, Liu B, Ji Y, Song X, Lv C, Zhang S. C-phycocyanin reinforces autophagy to block pulmonary fibrogenesis by inhibiting lncIAPF biogenesis. Arch Pharm Res. 2024;47:659–74. 39039254 10.1007/s12272-024-01508-y PMC11300487

[10] Li M, Li J, Wang Y, Jiang G, Jiang H, Li M, et al. Umbilical cord-derived mesenchymal stem cells preferentially modulate macrophages to alleviate pulmonary fibrosis. Stem Cell Res. 2024;15:475. 10.1186/s13287-024-04091-7 PMC11657361 39696548

[11] Tang Z, Gao J, Wu J, Zeng G, Liao Y, Song Z, et al. Human umbilical cord mesenchymal stromal cells attenuate pulmonary fibrosis via regulatory T cell through interaction with macrophage. Stem Cell Res. 2021;12:397. 10.1186/s13287-021-02469-5 PMC8278716 34256845

[12] Li M, Huang H, Wei X, Li H, Li J, Xie B, Yang Y, Fang X, Wang L, Zhang X, et al. Clinical investigation on nebulized human umbilical cord MSC-derived extracellular vesicles for pulmonary fibrosis treatment. Signal Transduct Target Ther. 2025;10:179. 40461474 10.1038/s41392-025-02262-3 PMC12134356

[13] Lan Y-W, Choo K-B, Chen C-M, Hung T-H, Chen Y-B, Hsieh C-H, Kuo H-P, Chong K-Y. Hypoxia-preconditioned mesenchymal stem cells attenuate bleomycin-induced pulmonary fibrosis. Stem Cell Res Ther. 2015;6:97. 25986930 10.1186/s13287-015-0081-6 PMC4487587

[14] Katzen J, Beers MF. Contributions of alveolar epithelial cell quality control to pulmonary fibrosis. J Clin Invest. 2020;130:5088–99. 32870817 10.1172/JCI139519 PMC7524463

[15] Bueno M, Calyeca J, Rojas M, Mora AL. Mitochondria dysfunction and metabolic reprogramming as drivers of idiopathic pulmonary fibrosis. Redox Biol. 2020;33:101509. 32234292 10.1016/j.redox.2020.101509 PMC7251240

[16] Wang Q, Xie Z-L, Wu Q, Jin Z-X, Yang C, Feng J. Role of various imbalances centered on alveolar epithelial cell/fibroblast apoptosis imbalance in the pathogenesis of idiopathic pulmonary fibrosis. Chin Med J. 2021;134:261. 33522725 10.1097/CM9.0000000000001288 PMC7846426

[17] Liu Z, Sun Y, Qi Z, Cao L, Ding S. Mitochondrial transfer/transplantation: an emerging therapeutic approach for multiple diseases. Cell Biosci. 2022;12:66. 35590379 10.1186/s13578-022-00805-7 PMC9121600

[18] Huang T, Lin R, Su Y, Sun H, Zheng X, Zhang J, Lu X, Zhao B, Jiang X, Huang L, et al. Efficient intervention for pulmonary fibrosis via mitochondrial transfer promoted by mitochondrial biogenesis. Nat Commun. 2023;14:5781. 37723135 10.1038/s41467-023-41529-7 PMC10507082

[19] Yuan J, Chen F, Jiang D, Xu Z, Zhang H, Jin Z-B. ROCK inhibitor enhances mitochondrial transfer via tunneling nanotubes in retinal pigment epithelium. Theranostics. 2024;14:5762–77. 39346535 10.7150/thno.96508 PMC11426248

[20] Borcherding N, Brestoff JR. The power and potential of mitochondria transfer. Nature. 2023;623:283–91. 37938702 10.1038/s41586-023-06537-z PMC11590279

[21] Wang L, Yang C, Wang Q, Liu Q, Wang Y, Zhou J, Li Y, Tan Y, Kang C. Homotrimer cavin1 interacts with caveolin1 to facilitate tumor growth and activate microglia through extracellular vesicles in glioma. Theranostics. 2020;10:6674–94. 32550897 10.7150/thno.45688 PMC7295042

[22] Li D, Jin Y, He X, Deng J, Lu W, Yang Z, Zheng X, Hou K, Tang S, Bao B, et al. Hypoxia-induced LAMB2-enriched extracellular vesicles promote peritoneal metastasis in gastric cancer via the ROCK1-CAV1-Rab11 axis. Oncogene. 2024;43:2768–80. 39138263 10.1038/s41388-024-03124-y

[23] Luo X, Zeng W, Xiong E, Wang Z, Huang J, Luo M, et al. Hypoxia-preconditioning human bone marrow-derived mesenchymal stem cells induce high-quality mitochondrial transfer through gap junctions to alleviate ischemia-reperfusion injury in liver graft. Cell Commun Signal. 2025;23:495. 41233822 10.1186/s12964-025-02497-1 PMC12613725

[24] Wei B, Ji M, Lin Y, Wang S, Liu Y, Geng R, et al. Mitochondrial transfer from bone mesenchymal stem cells protects against tendinopathy both in vitro and in vivo. Stem Cell Res Ther. 2023;14:104. 37101277 10.1186/s13287-023-03329-0 PMC10134653

[25] Zhang L, Ren C, He M, Chen X, Liu H, Liu Y, et al. Mitochondrial transfer in the HSC-HCC-macrophage network shapes hepatocellular carcinoma progression. Proc Natl Acad Sci U S A. 2026;123:e2512592123. 41642981 10.1073/pnas.2512592123 PMC12891106

[26] Zhang H, Zhu Q, Ji Y, Wang M, Zhang Q, Liu W, Li R, Zhang J, Xu P, Song X, Lv C. hucMSCs treatment prevents pulmonary fibrosis by reducing circANKRD42-YAP1-mediated mechanical stiffness. Aging. 2023;15:5514–34. 37335082 10.18632/aging.204805 PMC10333056

[27] Zhao L, Tang Y, Wu B, Gao Y, Shen X, Liu Z, Xiao S, Yao S, Li J, Shen F. Human umbilical cord mesenchymal stem cell-derived exosomes prevent and alleviate experimental pulmonary fibrosis by modulating the cytoskeleton of macrophages. Stem Cell Res Ther. 2025;16:533. 41024236 10.1186/s13287-025-04646-2 PMC12482893

[28] Zhao J, Jiang Q, Xu C, Jia Q, Wang H, Xue W, Wang Y, Zhu Z, Tian L. MiR-26a-5p from HucMSC-derived extracellular vesicles inhibits epithelial mesenchymal transition by targeting Adam17 in silica-induced lung fibrosis. Ecotoxicol Environ Saf. 2023;257:114950. 37099959 10.1016/j.ecoenv.2023.114950

[29] Zhang WY, Wen L, Du L, Liu TT, Sun Y, Chen YZ, Lu YX, Cheng XC, Sun HY, Xiao FJ, Wang LS. S-RBD-modified and miR-486-5p-engineered exosomes derived from mesenchymal stem cells suppress ferroptosis and alleviate radiation-induced lung injury and long-term pulmonary fibrosis. J Nanobiotechnol. 2024;22:662. 10.1186/s12951-024-02830-9 PMC11515248 39462403

[30] Li Z, Niu S, Guo B, Gao T, Wang L, Wang Y, Wang L, Tan Y, Wu J, Hao J. Stem cell therapy for COVID-19, ARDS and pulmonary fibrosis. Cell Prolif. 2020;53:e12939. 33098357 10.1111/cpr.12939 PMC7645923

[31] Zhao X, Kong C, Xia X, Zhao K, Hao M, Gao Y, Wang Y, Chen Z, Jiang J. Analysis of the therapeutic effect and potential mechanism of Fe(3)O(4)-polydopamine nanoparticles in enhancing mesenchymal stem cells therapy for pulmonary fibrosis. Mater Today Bio. 2025;34:102098. 40735701 10.1016/j.mtbio.2025.102098 PMC12305185

[32] Nwosu ZC, Ebert MP, Dooley S, Meyer C. Caveolin-1 in the regulation of cell metabolism: A cancer perspective. Mol Cancer. 2016;15:71. 27852311 10.1186/s12943-016-0558-7 PMC5112640

[33] Aboy-Pardal MCM, Guadamillas MC, Guerrero CR, Català-Montoro M, Toledano-Donado M, Terrés-Domínguez S, Pavón DM, Jiménez-Jiménez V, Jimenez-Carretero D, Zamai M, et al. Plasma membrane remodeling determines adipocyte expansion and mechanical adaptability. Nat Commun. 2024;15:10102. 39609408 10.1038/s41467-024-54224-y PMC11605069

[34] Li Y, Wen Y, Li Y, Tan X, Gao S, Fan P, Tian W, Wong CCL, Chen Y. Rab10-CAV1 mediated intraluminal vesicle transport to migrasomes. Proc. Natl. Acad. Sci. USA 2024; 121:e2319267121. 10.1073/pnas.2319267121 PMC11287133 39008679

[35] Sotodosos-Alonso L, Pulgarin-Alfaro M, Del Pozo MA. Caveolae Mechanotransduction at the Interface between Cytoskeleton and Extracellular Matrix. Cells 2023; 12: 44. 10.3390/cells12060942 PMC10047380 36980283

[36] Ni K, Wang C, Carnino JM, Jin Y. The Evolving Role of Caveolin-1: A Critical Regulator of Extracellular Vesicles. Med Sci (Basel) 2020; 8:45. 10.3390/medsci8040046 PMC7712126 33158117

[37] Sohn J, Lin H, Fritch MR, Tuan RS. Influence of cholesterol/caveolin-1/caveolae homeostasis on membrane properties and substrate adhesion characteristics of adult human mesenchymal stem cells. Stem Cell Res Ther. 2018;9:86. 29615119 10.1186/s13287-018-0830-4 PMC5883280

[38] Tang W, Yan C, He S, Du M, Cheng B, Deng B, Zhu S, Li Y, Wang Q. Neuron-targeted overexpression of caveolin-1 alleviates diabetes-associated cognitive dysfunction via regulating mitochondrial fission-mitophagy axis. Cell Commun Signal. 2023;21:357. 38102662 10.1186/s12964-023-01328-5 PMC10722701

[39] Cha K-Y, Cho W, Park S, Ahn J, Park H, Baek I, Lee M, Lee S, Arai Y, Lee S-H. Generation of bioactive MSC-EVs for bone tissue regeneration by tauroursodeoxycholic acid treatment. J Control Release. 2023;354:45–56. 36586671 10.1016/j.jconrel.2022.12.053

[40] Liang J, Huang G, Liu X, Zhang X, Rabata A, Liu N, Fang K, Taghavifar F, Dai K, Kulur V, et al. Lipid deficiency contributes to impaired alveolar progenitor cell function in aging and idiopathic pulmonary fibrosis. Am J Respir Cell Mol Biol. 2024;71:242–53. 38657143 10.1165/rcmb.2023-0290OC PMC11299087

[41] Panagiotidis G-D, Vasquez-Pacheco E, Chu X, Seeger W, El Agha E, Bellusci S, Lingampally A. Revisiting pulmonary fibrosis: Inflammatory dynamics of the lipofibroblast-to-inflammatory lipofibroblast-to-activated myofibroblast reversible switch. Front Immunol. 2025;16:1609509. 40607394 10.3389/fimmu.2025.1609509 PMC12213413

[42] Cai G, Liu J, Cai M, Shao L. Exploring the causal effect between lipid-modifying drugs and idiopathic pulmonary fibrosis: A drug-target mendelian randomization study. Lipid Health Dis. 2024;23:237. 10.1186/s12944-024-02218-6 PMC11293199 39090671

[43] Chen R, Dai J. Lipid metabolism in idiopathic pulmonary fibrosis: From pathogenesis to therapy. J Mol Med (Berl). 2023;101:905–15. 37289208 10.1007/s00109-023-02336-1

[44] Rajesh R, Atallah R, Barnthaler T. Dysregulation of metabolic pathways in pulmonary fibrosis. Pharmacol Ther. 2023;246:108436. 37150402 10.1016/j.pharmthera.2023.108436

[45] Dai T, Liang Y, Li X, Zhao J, Li G, Li Q, Xu L, Zhao J. Targeting alveolar epithelial cell metabolism in pulmonary fibrosis: Pioneering an emerging therapeutic strategy. Front Cell Dev Biol. 2025;13:1608750. 40636669 10.3389/fcell.2025.1608750 PMC12238887

[46] Shi X, Chen Y, Shi M, Gao F, Huang L, Wang W, Wei D, Shi C, Yu Y, Xia X, et al. The novel molecular mechanism of pulmonary fibrosis: Insight into lipid metabolism from reanalysis of single-cell RNA-seq databases. Lipid Health Dis. 2024;23:98. 10.1186/s12944-024-02062-8 PMC10988923 38570797

[47] Zhang Y, Li T, Pan M, Wang W, Huang W, Yuan Y, Xie Z, Chen Y, Peng J, Li X, Meng Y. SIRT1 prevents cigarette smoking-induced lung fibroblasts activation by regulating mitochondrial oxidative stress and lipid metabolism. J Transl Med. 2022;20:222. 35568871 10.1186/s12967-022-03408-5 PMC9107262

[48] Tong Z, Du X, Zhou Y, Jing F, Ma J, Feng Y, Lou S, Wang Q, Dong Z. Drp1-mediated mitochondrial fission promotes pulmonary fibrosis progression through the regulation of lipid metabolic reprogramming by ROS/HIF-1α. Cell Signal. 2024;117:111075. 38311302 10.1016/j.cellsig.2024.111075

[49] Yang J, Pan X, Xu M, Li Y, Liang C, Liu L, Li Z, Wang L, Yu G. Downregulation of HMGCS2 mediated AECIIs lipid metabolic alteration promotes pulmonary fibrosis by activating fibroblasts. Respir Res. 2024;25:176. 38658970 10.1186/s12931-024-02816-z PMC11040761

[50] Wang L, Yuan H, Li W, Yan P, Zhao M, Li Z, Zhao H, Wang S, Wan R, Li Y, et al. ACSS3 regulates the metabolic homeostasis of epithelial cells and alleviates pulmonary fibrosis. Biochim. Biophys. Acta (BBA) Mol. Basis Dis. 2024;1870:166960. 10.1016/j.bbadis.2023.166960 37979225

