# Supplementary material for: CAV1-dependent mitochondrial transfer from hucMSCs reprograms epithelial lipid metabolism to relieve pulmonary fibrosis
Source: Stem Cell Res Ther. 2026 Jun 19;17:307. doi: 10.1186/s13287-026-05116-z (PMC13548484; doi:10.1186/s13287-026-05116-z)

fig. 2 C

SPC

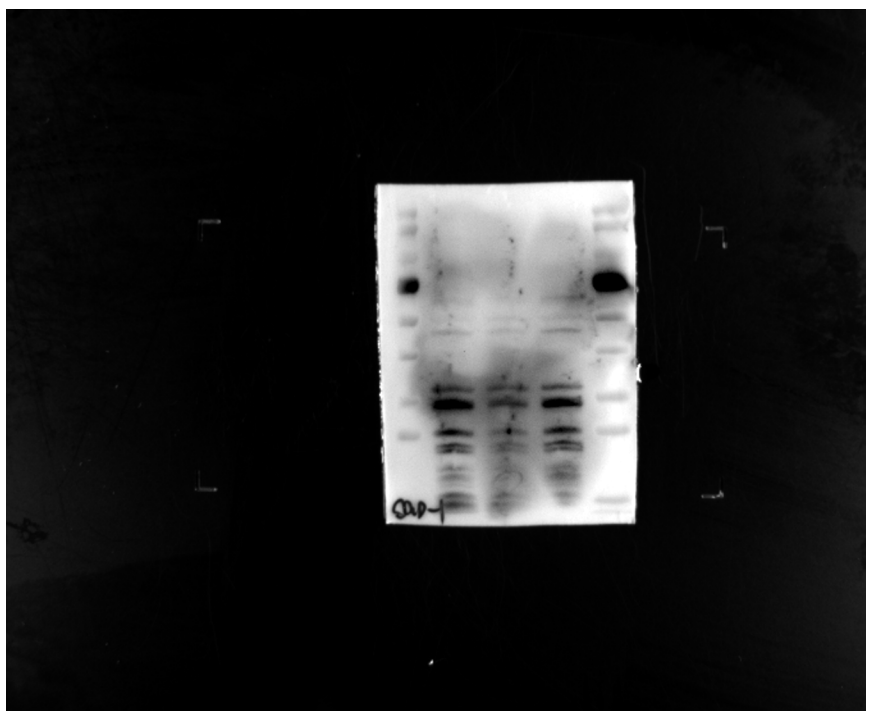

GAPDH

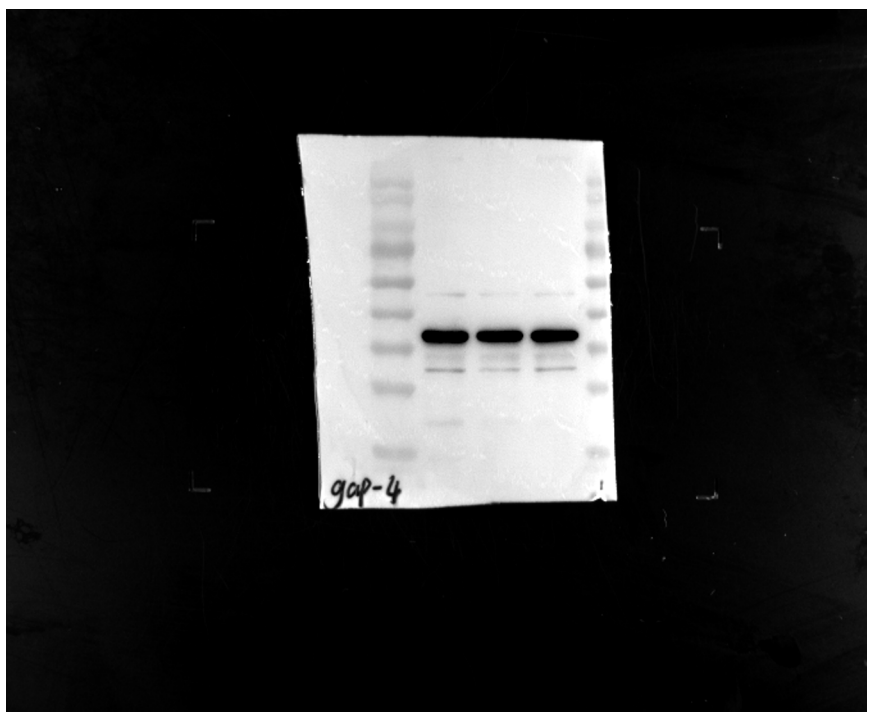

fig. 2 J

Collagen

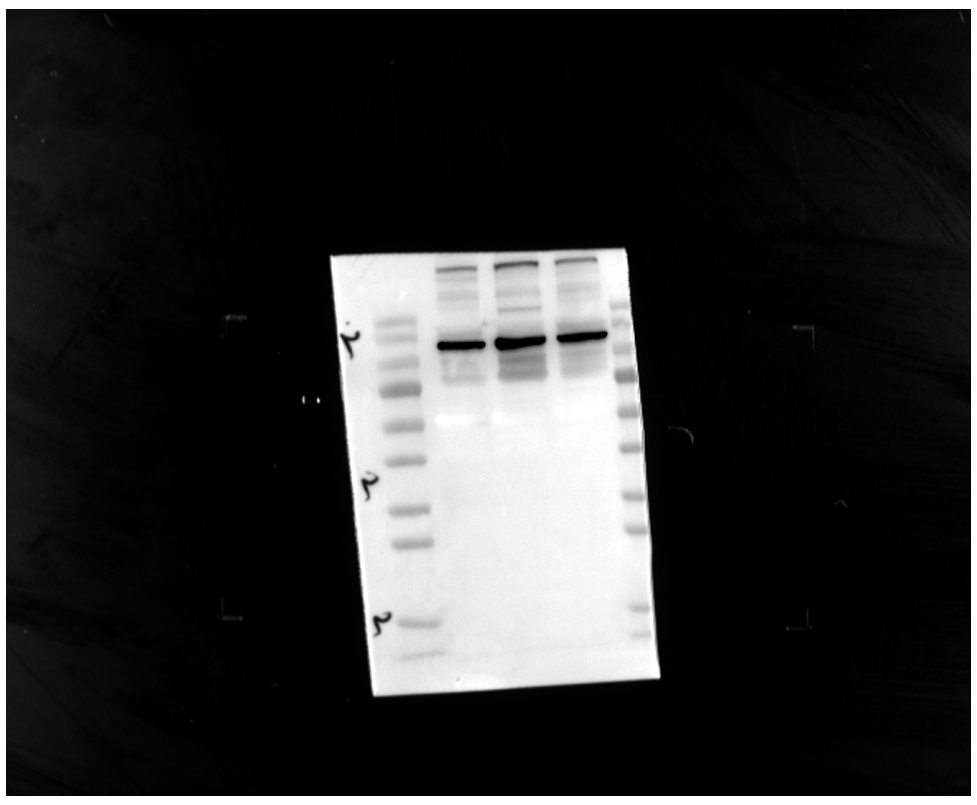

Vimentin

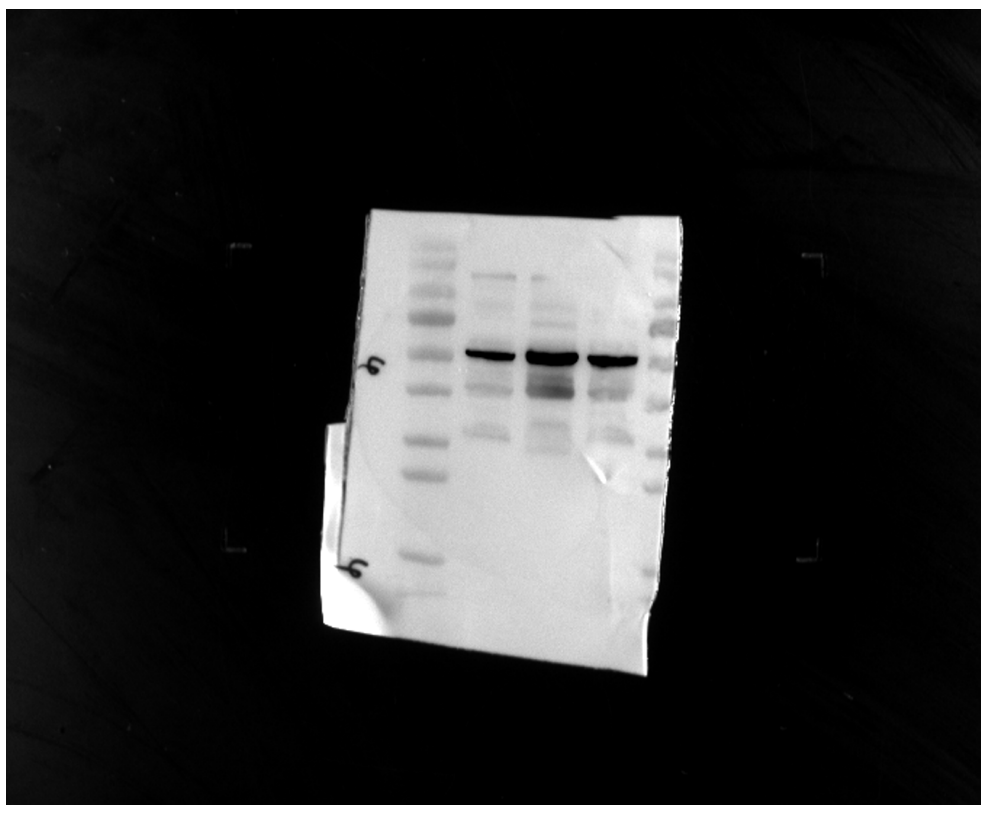

α-SMA

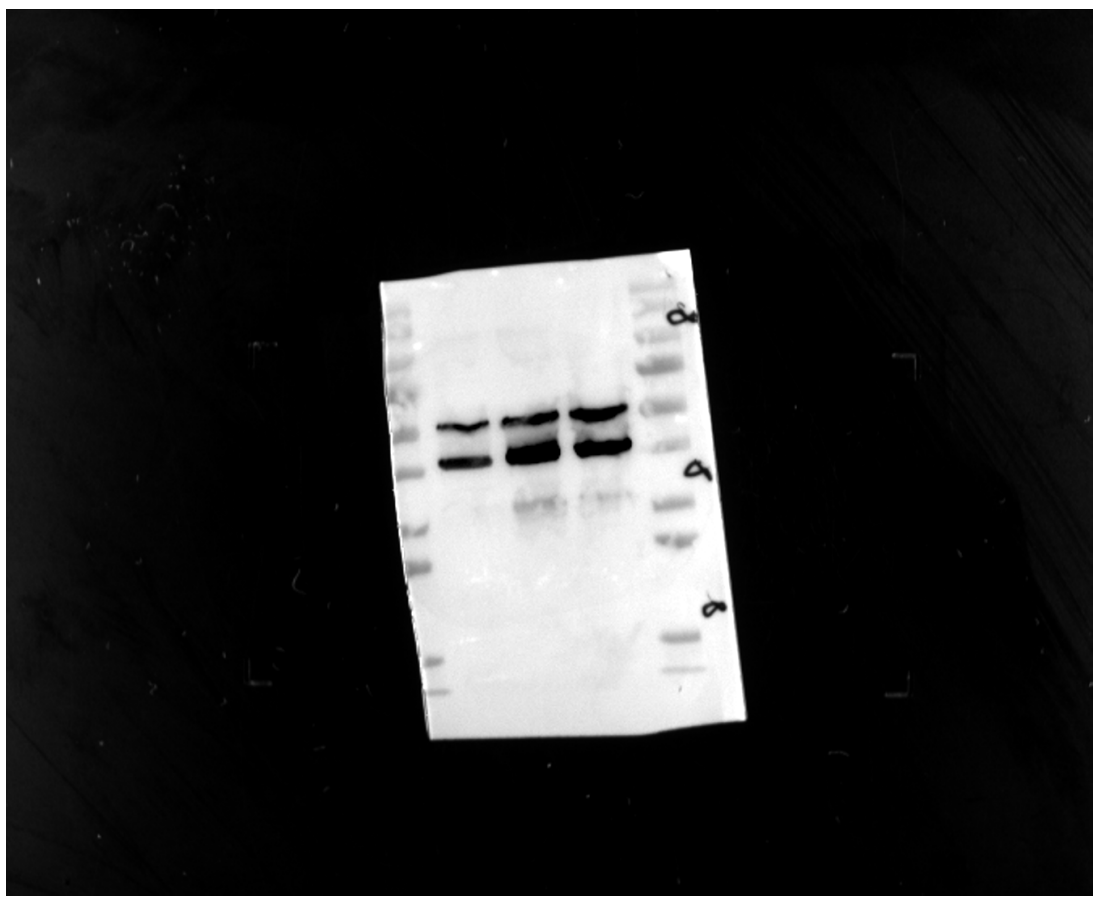

GAPDH

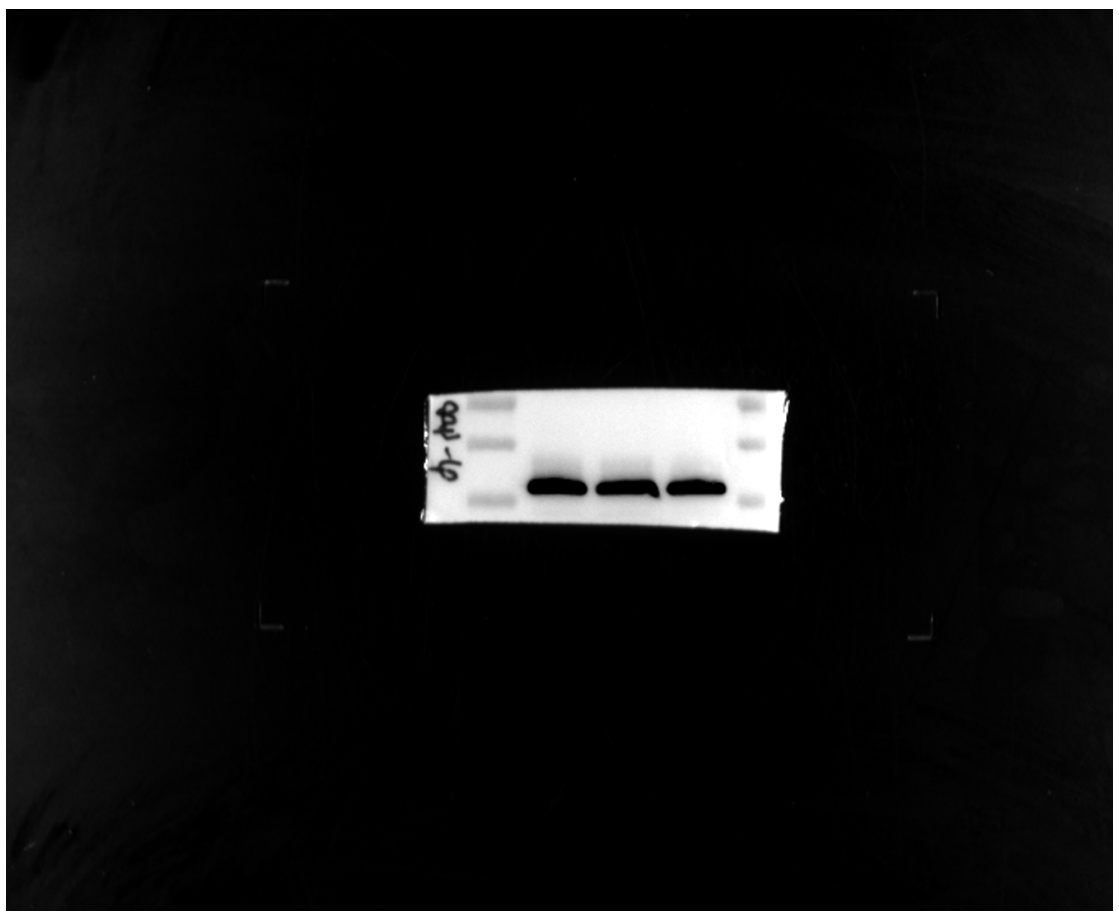

fig. 2 K

SPC

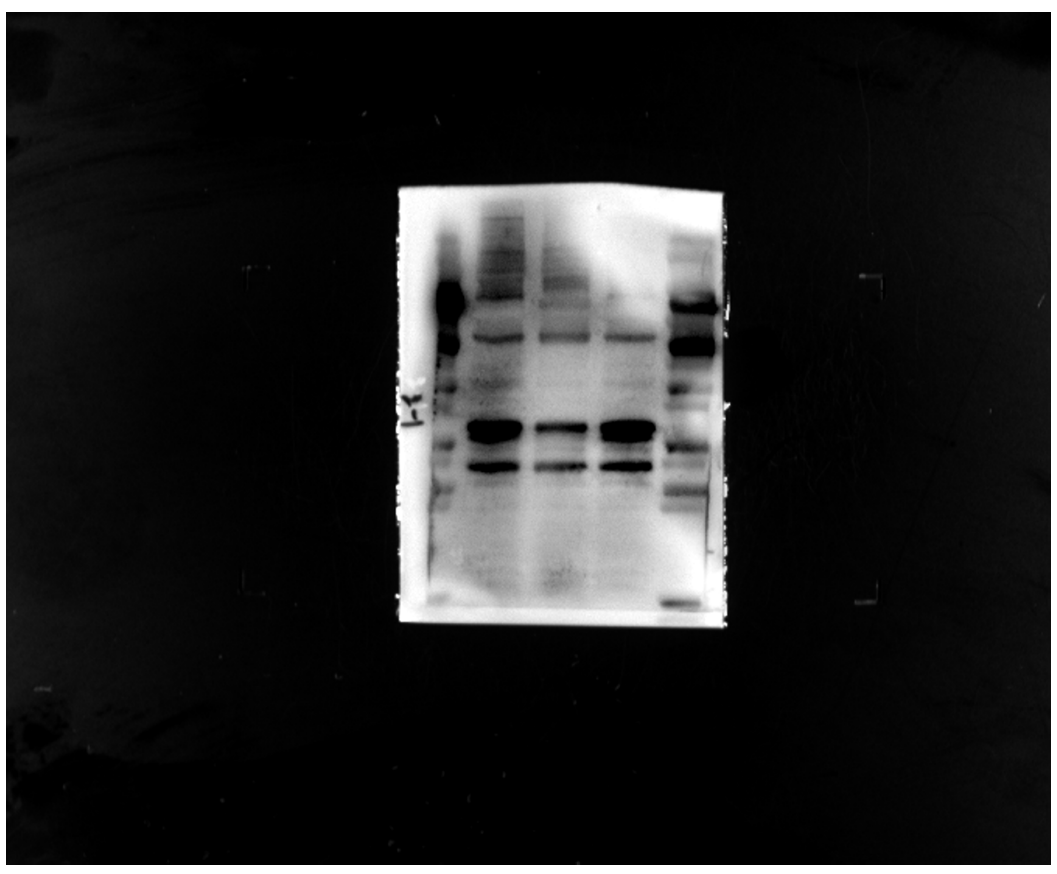

GAPDH

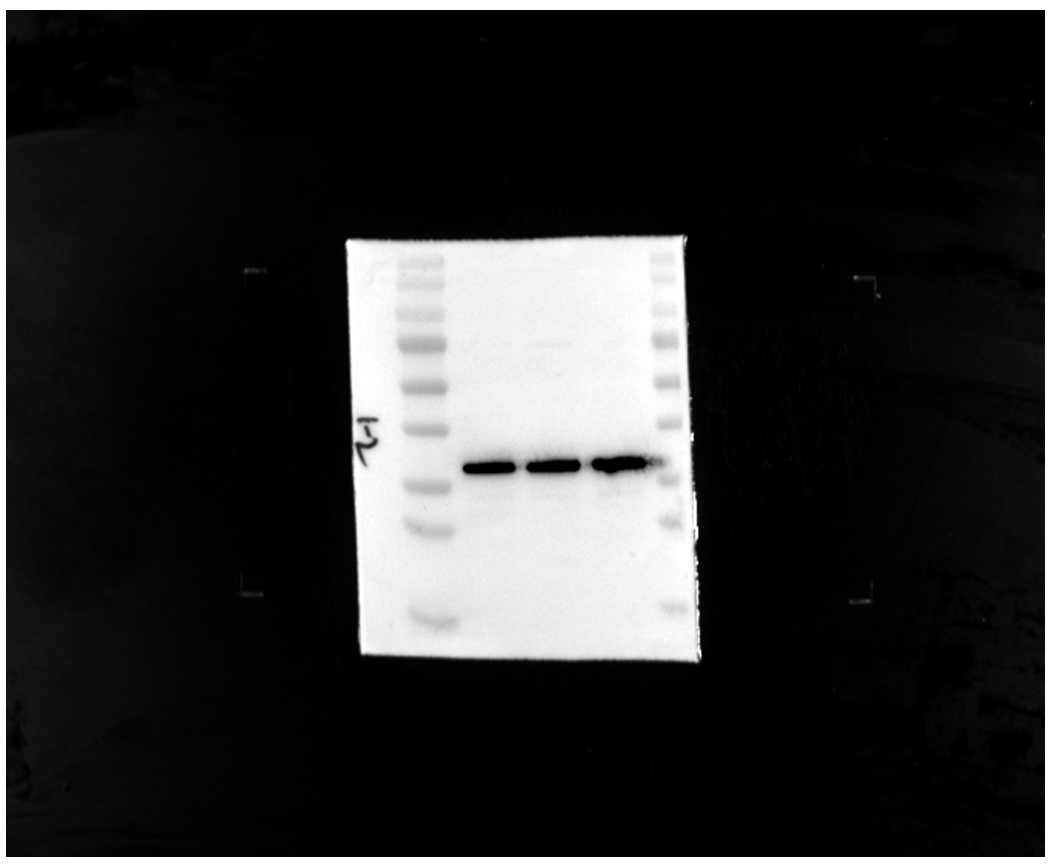

Supplement: Supplementary file 1 [file 13287_2026_5116_MOESM1_ESM.pdf]
