# Supplementary material for: CAV1-dependent mitochondrial transfer from hucMSCs reprograms epithelial lipid metabolism to relieve pulmonary fibrosis
Source: Stem Cell Res Ther. 2026 Jun 19;17:307. doi: 10.1186/s13287-026-05116-z (PMC13548484; doi:10.1186/s13287-026-05116-z)
Supplement: Supplementary file 4 [file 13287_2026_5116_MOESM6_ESM.docx]

**Supplementary Fig. S1 hucMSCs restore mitochondrial function in injured epithelial cells and this effect is attenuated under Transwell conditions.**

**A** JC-1 staining analysis of mitochondrial membrane potential (MMP) in MLE-12 cells. Representative flow cytometry plots and quantification showed that MMP was significantly decreased in BLM-treated cells and was partially restored after hucMSC treatment. **B** Flow cytometry analysis of ROS levels using DCFH-DA staining. ROS levels were markedly elevated in BLM-treated cells and were reduced following hucMSC treatment. Quantification is shown as relative mean fluorescence intensity (MFI). **C** ATP production in MLE-12 cells. BLM treatment significantly decreased ATP levels, which were restored after hucMSC treatment.

**Supplementary Fig. S2**

**Supplementary Fig. S2 Endogenous CAV1 expression is increased in hucMSCs after coculture with BLM-injured MLE-12 cells.** Western blot analysis showing endogenous CAV1 protein expression in hucMSCs under normal culture conditions and after coculture with BLM-injured MLE-12 cells. GAPDH was used as the loading control. The results confirmed that CAV1 expression was increased in cocultured hucMSCs compared with normal hucMSCs.

**Supplementary Fig. S3**

**Supplementary Fig. S3 | Flow cytometry gating strategy used for mitochondrial transfer and cell sorting.**
(**A**) Single-color controls of ZsGreen-MLE-12 and mtDsRed-hucMSCs were used to establish fluorescence boundaries and compensation. (**B**) Representative distribution of cocultured cells. (**C**) ZsGreen-positive cells were gated as recipient MLE-12 epithelial cells. Mitochondrial transfer was quantified by detecting mtDsRed fluorescence within the ZsGreen-positive population. (**D**) ZsGreen-negative cells were gated as hucMSCs.
**(E)** Negative control showing the unlabeled coculture of MLE-12 cells and hucMSCs, used to define the ZsGreen-negative background. **(F)** Sorting strategy for fluorescence-based downstream assays, in which unlabeled MLE-12 cells were cocultured with ZsGreen-labeled hucMSCs and ZsGreen-negative cells were collected as the MLE-12 population to avoid fluorescence interference.

**Supplementary Fig. S4**

**Supplementary Fig. S4** **Validation of mitochondrial labeling** (A) Confocal images showing co-localization of DsRed-labeled mitochondria with MitoTracker Green in hucMSCs, confirming specific mitochondrial labeling.
(B) ZsGreen (green) is localized in the cytoplasm and does not overlap with MitoTracker Red–labeled mitochondria (red).

**Supplementary Fig. S5 In vivo tracking of transplanted hucMSCs in lung tissue.**
Frozen lung sections showed no DiI signal in the BLM group, whereas DiI-labeled hucMSCs were detectable in the lung on both day 3 and day 7 after intravenous infusion, indicating pulmonary localization and short-term retention in vivo.
